# Multi-functional mechanisms of immune evasion by the streptococcal complement inhibitor C5a peptidase

**DOI:** 10.1371/journal.ppat.1006493

**Published:** 2017-08-14

**Authors:** Nicola N. Lynskey, Mark Reglinski, Damien Calay, Matthew K. Siggins, Justin C. Mason, Marina Botto, Shiranee Sriskandan

**Affiliations:** Faculty of Medicine, Imperial College London, London, United Kingdom; New York Medical College, UNITED STATES

## Abstract

The complement cascade is crucial for clearance and control of invading pathogens, and as such is a key target for pathogen mediated host modulation. C3 is the central molecule of the complement cascade, and plays a vital role in opsonization of bacteria and recruitment of neutrophils to the site of infection. Streptococcal species have evolved multiple mechanisms to disrupt complement-mediated innate immunity, among which ScpA (C5a peptidase), a C5a inactivating enzyme, is widely conserved. Here we demonstrate for the first time that pyogenic streptococcal species are capable of cleaving C3, and identify C3 and C3a as novel substrates for the streptococcal ScpA, which are functionally inactivated as a result of cleavage 7 amino acids upstream of the natural C3 convertase. Cleavage of C3a by ScpA resulted in disruption of human neutrophil activation, phagocytosis and chemotaxis, while cleavage of C3 generated abnormally-sized C3a and C3b moieties with impaired function, in particular reducing C3 deposition on the bacterial surface. Despite clear effects on human complement, expression of ScpA reduced clearance of group A streptococci *in vivo* in wildtype and C5 deficient mice, and promoted systemic bacterial dissemination in mice that lacked both C3 and C5, suggesting an additional complement-independent role for ScpA in streptococcal pathogenesis. ScpA was shown to mediate streptococcal adhesion to both human epithelial and endothelial cells, consistent with a role in promoting bacterial invasion within the host. Taken together, these data show that ScpA is a multi-functional virulence factor with both complement-dependent and independent roles in streptococcal pathogenesis.

## Author summary

The complement pathway is critical in the innate immune response to bacterial pathogens. It consists of a self-perpetuating proteolytic cascade initiated via three distinct pathways that converge at the central complement protein, C3. Pathogens must evade complement-mediated immunity to cause disease, and inactivation of the C3 protein can dampen all effectors of this pathway. Streptococcal species are the causative agents of an array of infections ranging from the benign to lethal. Using the human pathogen Group A *Streptococcus* as a representative species, we show that the enzyme ScpA, which is conserved amongst the pyogenic streptococci, cleaves human C3a and also C3, releasing abnormally sized and functionally-impaired fragments. As a result, invading streptococci were less well opsonized and host immune cells not properly activated, reducing bacterial phagocytosis and clearance. Despite manifest *in vitro* activity against complement factors and human neutrophils, ScpA was still able to contribute to systemic bacterial spread in mice lacking C3 and C5. ScpA was also demonstrated to mediate streptococcal adhesion to both epithelial and endothelial cells, which may enhance bacterial systemic spread. Our study highlights the likely importance of both complement-independent and complement-dependent roles for ScpA in streptococcal pathogenesis.

## Introduction

The complement cascade is crucial for clearance of invading pathogens and thus represents a key target for disruption by such organisms. Individual bacterial species use multiple strategies to escape the complement system, highlighting the importance of this pathway in bacterial immunity [1–7]. The human pathogen Group A *Streptococcus* (GAS), the causative agent of over half a million infections globally each year which range from benign to life-threatening, is no exception [8]. The ability of GAS to successfully colonize the host and resist clearance is mediated by an array of virulence factors, a number of which interfere with and inactivate the complement cascade [2,4,9–11].

The complement pathway comprises a tightly regulated, self-perpetuating proteolytic cascade that results in clearance of pathogens by a combination of opsonization, anaphylatoxin release and formation of the lytic membrane attack complex (MAC). Activation occurs via three routes; classical, alternative and lectin pathways, all of which converge at the central complement component C3 [12], a 186 kDa member of the α-macroglobulin family [13].

C3 is comprised of an α (111 kDa) and β (75 kDa) chain which are linked by multiple disulfide bonds. C3 function is modulated by a sequence of proteolytic events, the first of which is mediated by the C3 convertase, and results in the release of the 9 kDa anaphylatoxin C3a from the N-terminus of the C3 α-chain. Activity of C3a is dependent on the carboxy-terminus of the protein, and is quenched following cleavage of the C-terminal arginine residue by the carboxypeptidase B enzyme [13]. The remaining 177 kDa protein, C3b, comprises the residual 102 kDa α–chain and 75 kDa β–chain, and is the activated form of C3. Conformational changes following cleavage result in exposure of a reactive thioester residue permitting covalent deposition on the bacterial surface. Bound C3b interacts with complement receptors expressed by circulating phagocytes, mediating bacterial uptake and killing.

C3b also binds to the pro-enzyme Factor B, cleavage of which by Factor D results in formation of the enzyme complex C3bBb. C3bBb catalyzes cleavage of C3 to C3b and C3a thus amplifying the complement response. [12] This activity in turn induces formation of the C3b_2_Bb complex which cleaves the complement component C5. C5 is also a member of the α-macroglobulin family and as such shares a similar structure to C3. C5 is cleaved by C3b_2_Bb which results in the release of the 10.4 kDa anaphylatoxin C5a from the N-terminus similar to the release of C3a from C3. The remaining, larger C5b protein is necessary for downstream complement activation [13].

While deposition of antibody on the surface of GAS is important in promoting opsonophagocytosis [14], complement deposition plays a pivotal role in the control of GAS infection. A strong selective pressure to resist complement immunity has resulted in the evolution of numerous evasion strategies in GAS [2, 4, 9–11]. These include expression of virulence factors that bind to and inactivate key components of the complement cascade and/or sequester negative regulators of complement at the bacterial surface. GAS also produces enzymes that cleave and inactivate complement components. These include the promiscuous secreted protease SpeB, which degrades numerous host factors including C3b [4,11], and the C5a-inactivating serine protease ScpA, that is common to many pathogenic streptococci, which specifically cleaves C5a [10,15,16,17].

ScpA is a member of the subtilisin-like serine protease family, containing a highly conserved catalytic triad motif (Asp^130^, His^193^, Ser^512^) that is critical for enzymatic activity [16]. Additionally ScpA has an LPXTG motif at the C-terminus which permits anchoring of the protein to the bacterial cell wall [15]. ScpA is processed from a pre- to pro-peptide by autocatalytic cleavage of the N-terminal 31 amino acids, resulting in a catalytically active protein [10]. The reported substrate for ScpA is the human anaphylatoxin C5a, which plays a key role in neutrophil activation and recruitment to the site of infection. ScpA cleaves C5a at the His^67^ residue, releasing the C-terminus and rendering the protein inactive [10]. Thus, ScpA activity significantly impedes neutrophil activation and recruitment to the site of infection [17], and has been shown to promote bacterial persistence and dissemination in murine models of infection [17–19]. C5a is the only reported substrate for ScpA, which is surprising given the specificity of other members of this enzyme family [20].

Although GAS have been reported to bind to [2, 6, 7, 9] or inactivate [4, 11] numerous effectors of the complement cascade, they have not been reported to cleave the central molecule C3, inactivation of which would dampen all anti-bacterial effectors of this pathway. In a previous proteomic screen we identified a putative uncharacterized protein annotated as a “C3-degrading protease (CppA)” [21] which is conserved amongst several streptococal species [21]. This prompted us to question whether GAS and other pyogenic streptococci could actually specifically cleave the central complement component C3. Here, we report that Groups A, C and G streptococci are able to cleave C3, but that the phenotype was not mediated by CppA. Using GAS as a model organism, we went on to demonstrate that the complement-cleaving activity was mediated by ScpA. As such, we identified C3a and C3 as novel substrates for ScpA, cleavage of which is associated with reduced human neutrophil activation and chemotaxis, and subsequent reduced bacterial opsonophagocytosis and killing.

Expression of ScpA has previously been implicated in streptococcal pathogenesis in murine infection models [17–19]. Here, we demonstrate that ScpA confers resistance to bacterial clearance in a streptococcal soft-tissue infection model that was manifest even in mice lacking both C3 and C5. Thus, notwithstanding any inactivation of the human complement cascade, ScpA confers virulence to GAS in mice independently of C3 or C5 cleavage, a phenomenon that, we suggest, is likely to be dependent on ScpA-mediated attachment of GAS to endothelial and epithelial cells, as demonstrated *in vitro*. Overall, our study characterizes ScpA as a multi-functional, complement inactivating protein, with properties that confer pathogenicity at multiple stages of infection.

## Results

### Streptococcal species cleave human C3 in a conserved manner

**Fig 1:**
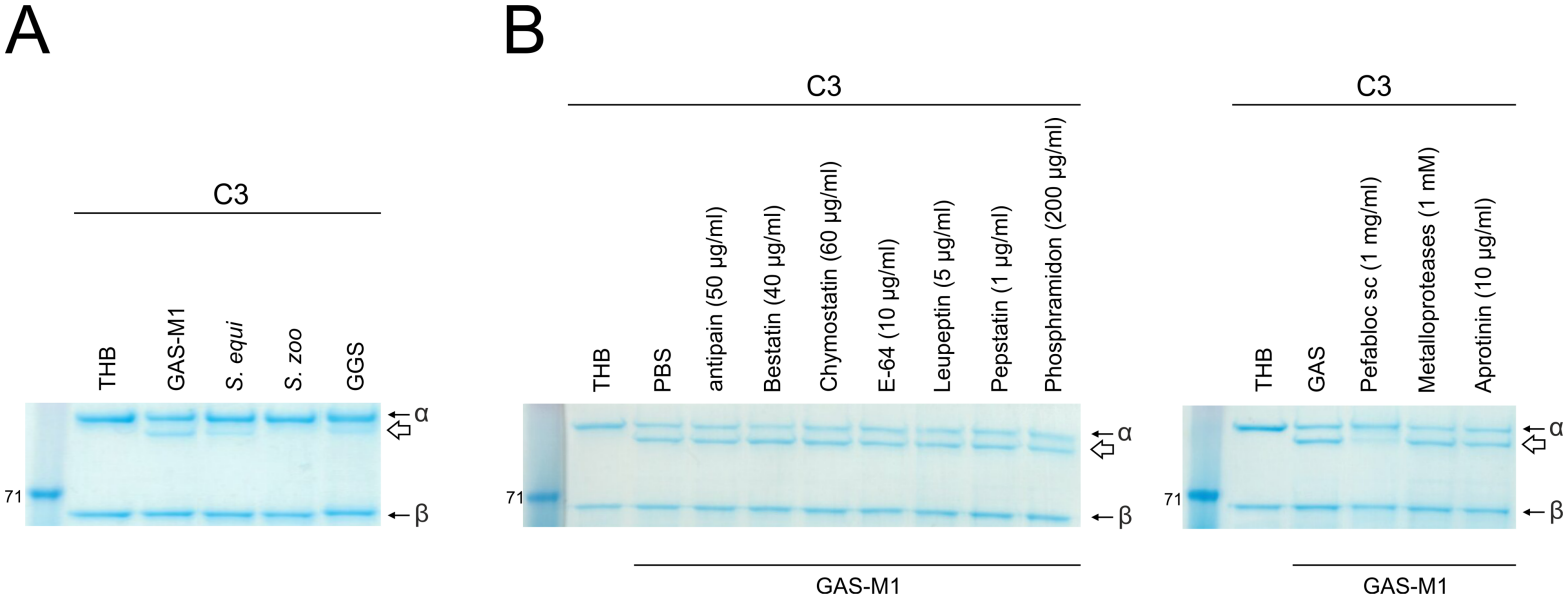
Streptococcal species cleave human C3. A) Cleavage of human C3 by representative strains of Groups A (GAS-M1), C (*S*. *zoo* and *S*. *equi*) and G (GGS) streptococci. Bacterial pellets (4x10^6^ cfu) were incubated with human C3 for 16 hours, 37°C. Specific cleavage of the C3α chain was visualized following SDS-PAGE and resulted in release of a 100 kDa product, C3α^scpA^ (white arrow). No cleavage of the β chain was observed. B) The impact of a panel of protease inhibitors on C3 cleavage by GAS-M1 cell pellets was assessed. GAS-M1 pellets (4x10^6^ cfu) were incubated with human C3 for 16 hours at 37°C in the presence of individual protease inhibitors and compared for ability to generate cleavage fragment C3α^scpA^ (white arrow). Concentration of each protease inhibitor used is indicated. The serine protease Pefabloc was sufficient to inhibit streptococcal cleavage of C3.

In a previous study we identified a putative “C3-degrading protease” CppA [21], for which homologues exist in other streptococcal species [22], and showed that it is upregulated in GAS lacking a functional version of the regulator RocA [21]. In an effort to determine whether streptococcal species could cleave C3, as has been demonstrated for *S*. *pneumoniae* [22], whole cell suspensions of streptococcal species representing Lancefield groups A, C and G were co-incubated with human C3. Human C3 is a 186 kDa protein comprised of a 111 kDa α chain and a 75 kDa β chain [13]. These fragments are linked by multiple disulphide bonds and as such, under reducing conditions, C3 migrates as two separate bands of corresponding size. Incubation of different streptococcal species with C3 generated an additional 100 kDa α-chain (C3α^scpA^) absent from controls incubated with buffer alone. This demonstrated that the experimental setting did not induce physiological activation of C3 to iC3b, which would result in release of a smaller α-chain, consistent with streptococcal-mediated cleavage of the alpha chain of C3 (Fig 1A).

Initially we carried out experiments to determine whether the CppA protein, identified in our previous study, was capable of cleaving C3. We were, however, unable to demonstrate any role for CppA in the observed cleavage of human C3, since recombinant CppA did not reproduce the activity of whole streptococcal preparations (S1 and S2A Figs). Furthermore, there was no evidence that the streptococcal cysteine protease SpeB, previously reported to degrade C3b [11], was responsible for cleavage of full-length C3. Whole bacterial cells from isogenic strains that differed only in production of active SpeB (GAS-M49 and GAS-M49_ΔscpA_) were both equally able to cleave C3, while supernatant fractions of the same strains were unable to cleave full-length C3 at all (S2B Fig).

In order to broadly characterize the type of enzyme mediating C3 cleavage, we tested a panel of 10 protease inhibitors to determine if any were sufficient to impair cleavage. Only the serine protease inhibitor Pefabloc was sufficient to inhibit cleavage of C3 by GAS-M1 (Fig 1B). GAS produce two serine proteases known to interact with the host immune response, the CXC-chemokine-cleaving enzyme SpyCEP [23] and ScpA. Isogenic strains that differed only in production of SpyCEP (GAS-M81 and GAS-M81_ΔspyCEP_), previously described by our laboratory [24] were equally able to cleave C3, ruling out SpyCEP as the C3-ase (S2C Fig). Thus in order to ascertain whether cleavage of C3 was mediated by ScpA, ScpA-negative isogenic strains were generated using two major GAS serotypes, M1 and M89, and extensively screened to ensure no polar effects on expression of adjacent Mga-regulated, or CovR/S regulated genes had been introduced (S3A–S3F Fig).

### ScpA cleaves human C3

**Fig 2:**
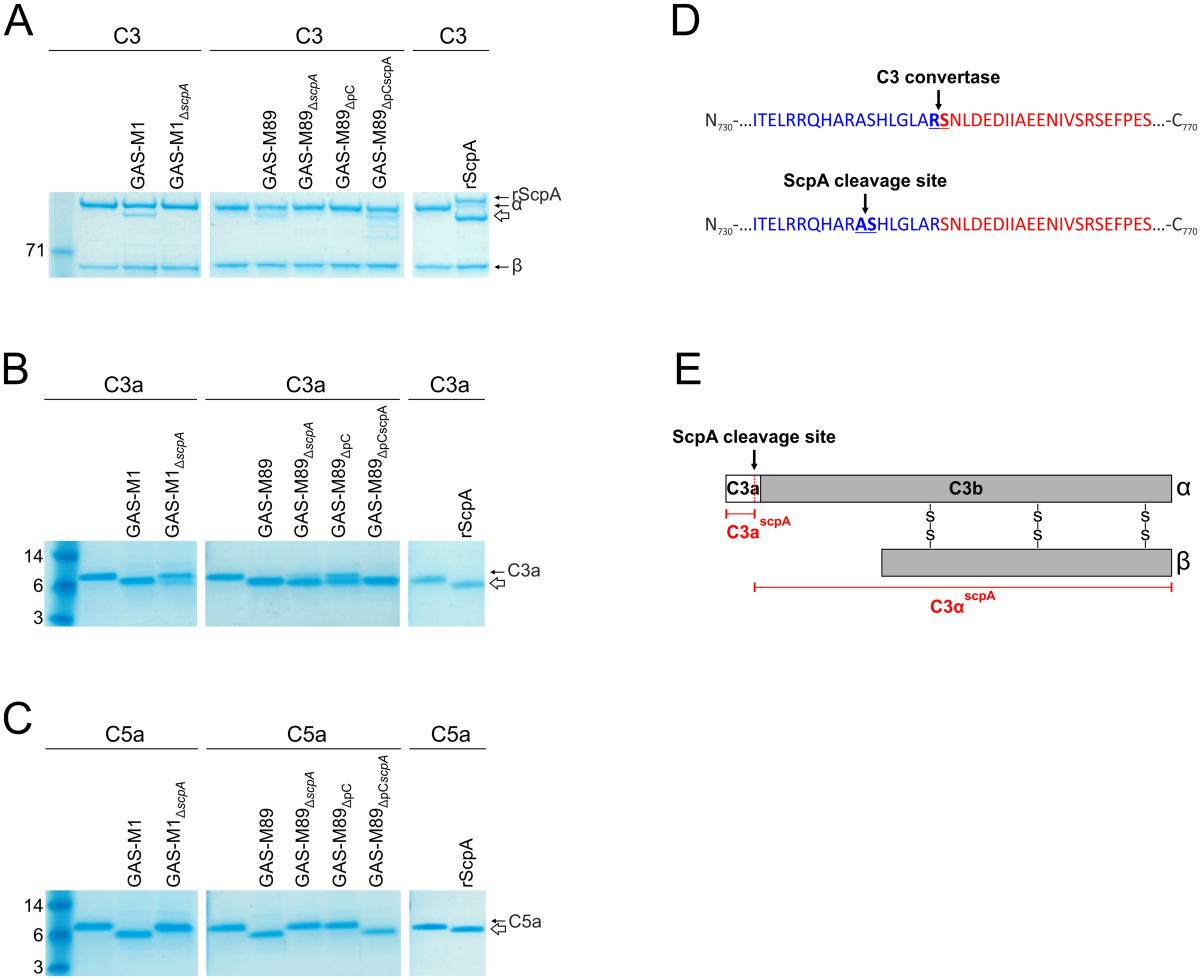
ScpA is necessary and sufficient for cleavage of human C3. A-C) Cleavage of human complement factors A) C3, B) C3a and C) C5a by whole cell pellets (4x10^6^ cfu) of GAS-M1 vs GAS-M1_ΔscpA_, GAS-M89 vs GAS-M89_ΔscpA_ and rScpA (100 ng). Only bacterial strains expressing ScpA were able to cleave these complement factors. Cleaved moieties (C3α^scpA^, C3a^scpA^, C5a^scpA^) are indicated by a white arrow in each panel. D) C3α^scpA^ was subjected to N-terminal sequencing. ScpA was demonstrated to cleave C3 between Ala^741^ and Ser^742^, 7aa N-terminal to the physiological C3 convertase cleavage site. E) Schematic representation of C3 α and β chains and the internal ScpA cleavage site. C3 is cleaved by C3 convertase to release C3b (grey bar) and C3a (white bar). The vertical black line between them shows the physiological C3 convertase cleavage site. ScpA cleaves C3 at an alternative site (red dotted line, black arrow) to release unique cleavage products, C3a^scpA^ and C3α^scpA^ (red text).

Wildtype strains GAS-M1 and GAS-M89 and the corresponding isogenic ScpA deletion mutants were investigated for C3 cleavage activity. As expected, 16 hour incubation of either wildtype strain with C3 yielded the additional 100 kDa C3α^scpA^ fragment consistent with cleavage; importantly, this band was absent following incubation with each of the mutants (Fig 2A). Cleavage of C3 was restored in GAS-M89_ΔscpA_ following complementation with plasmid pOri_scpA_ (Fig 2A). To verify that ScpA alone was mediating cleavage, the activity of recombinantly expressed ScpA (rScpA) (S1 Fig) was also evaluated. Similar to wildtype GAS, incubation of C3 with rScpA resulted in the release of a 100 kDa C3α^scpA^ fragment, confirming that C3 is a novel substrate for ScpA (Fig 2A).

The structural and functional homology shared between C3a and C5a are well reported in the literature [25]. It therefore seemed plausible that the C3a moiety may represent an additional substrate for ScpA, such that cleavage of both the inert N-terminus of full-length C3, comprising C3a, and the activated C3a fragment (released by host C3 convertase from C3) could occur. Incubation of wildtype GAS-M1 and GAS-M89 with human C3a resulted in a clear reduction in size when visualized by SDS-PAGE (Fig 2B), consistent with the size difference observed between the C3α and the C3α^scpA^ fragment of C3 (Fig 2A), and similar to the cleavage pattern observed for C5a (Fig 2C). Disruption of the *scpA* gene and complementation with plasmid pOri_scpA_ demonstrated that ScpA can cleave the C3a moiety, a finding that was confirmed by use of purified rScpA (Fig 2B). The cleavage of C3a we observed was not fully reversed by disruption of the *scpA* locus, suggesting that an additional as yet unknown GAS virulence factor may contribute to this effect (Fig 2B). Intriguingly, the ScpA-independent cleavage appeared more pronounced following incubation with GAS-M89_ΔscpA_ compared with GAS-M1_ΔscpA_, suggesting serotype variation in expression levels.

The pattern of cleavage provided evidence that the C3α-chain was being cleaved at the N terminus within C3a, and thus we carried out N-terminal sequencing of the C3α^scpA^ moiety following 16 hour incubation with rScpA to identify the ScpA cleavage site. The sequence obtained (_742-_SHLGL_-746_) indicated that ScpA cleaved C3α seven amino acids N-terminal to the site of physiological C3 convertase (Fig 2D and 2E). The ScpA cleavage site was two amino acids N-terminal to the cleavage site of NalP (S4 Fig), a C3-ase of *Neisseria meningitidis* that impairs complement-mediated immunity [26]. The disparity in size of ScpA-cleaved C3a (C3a^scpA^) and C3α (C3α^scpA^) compared with physiological counterparts C3a and C3b was greater than that reported for NalP, and thus pointed to a likely functional role for ScpA-mediated cleavage on C3 activity. Importantly other complement factors, C1q, C2 and the structurally similar C4, were not cleaved by GAS-M1 under the same conditions (S5 Fig), demonstrating the restricted substrate specificity of this enzyme.

**Fig 3:**
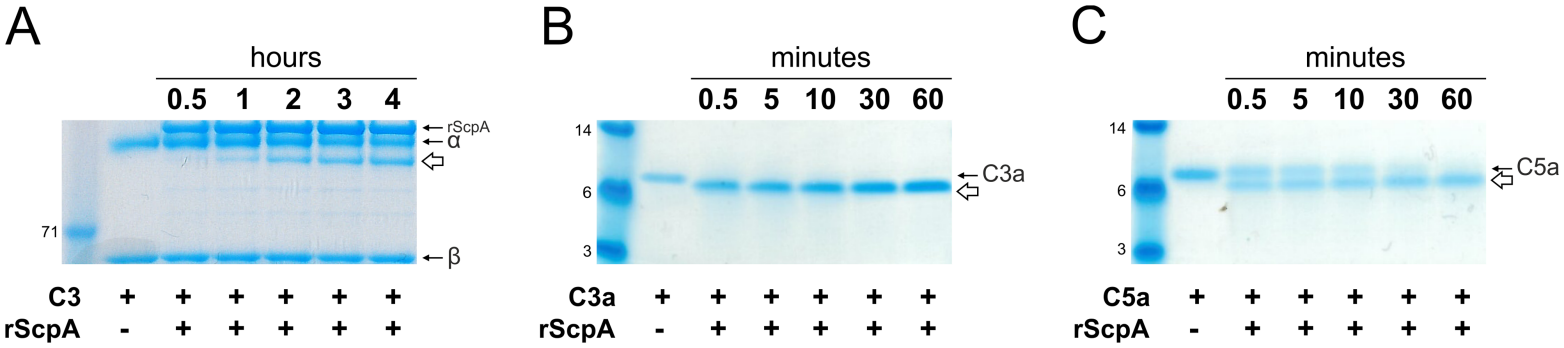
Rapid cleavage of complement factors by streptococcal ScpA. Rate of rScpA cleavage of complement components was assessed at 37°C and visualized by SDS-PAGE and Coomassie staining. A) Cleavage of C3 by rScpA at 1:1 molar ratio. C3α^scpA^ cleavage product (white arrow) was detectable after incubation with rScpA for 30 minutes, and became more pronounced by 60 minutes. B) Cleavage of C3a by rScpA at 10:1 molar ratio. C3a appeared to be completely cleaved to C3a^scpA^ (white arrow) following 0.5 minutes incubation with rScpA. C) Cleavage of C5a by rScpA at 10:1 molar ratio. C5a^scpA^ cleavage product (white arrow) was visible following 0.5 minutes incubation with rScpA. Complete cleavage was observed within 60 minutes.

To determine the functional relevance of C3-ase activity of ScpA, cleavage of human complement factors C3, C3a and C5a by rScpA protein were investigated over a time course (Fig 3). Cleavage of C3 by rScpA was tested at a 1:1 molar ratio over four hours, and release of C3α^scpA^ fragment was visualized by SDS-PAGE (Fig 3). Although cleavage of C3 required 30 minutes incubation with rScpA (Fig 3A), complete cleavage of C3a occurred within 30 seconds (Fig 3B). Cleavage of C3a occurred more rapidly than C5a (Fig 3C), suggesting that C3a inactivation may contribute to the phenotype previously attributed to C5a inactivation alone.

The staphylococcal cysteine protease Aureolysin (Aur) has previously been reported to cleave C3 when purified from *S*. *aureus* supernatants, with a recognized impact on complement function [12]. In order to directly compare rScpA protein function with Aur, we amplified *aur* from *S*. *aureus* strain N315 in order to express and purify rAur (S1 Fig). As described previously, rAur was only able to cleave C3 in HEPES++ reaction buffer [12], so comparative experiments with rScpA were carried out under these conditions rather than THB, as for earlier rScpA cleavage analyses. The rate of C3 cleavage was comparable for rScpA and rAur, and was visible from 30 minutes incubation (S6A Fig).

### ScpA cleavage is associated with impaired C3 opsonization of GAS

**Fig 4:**
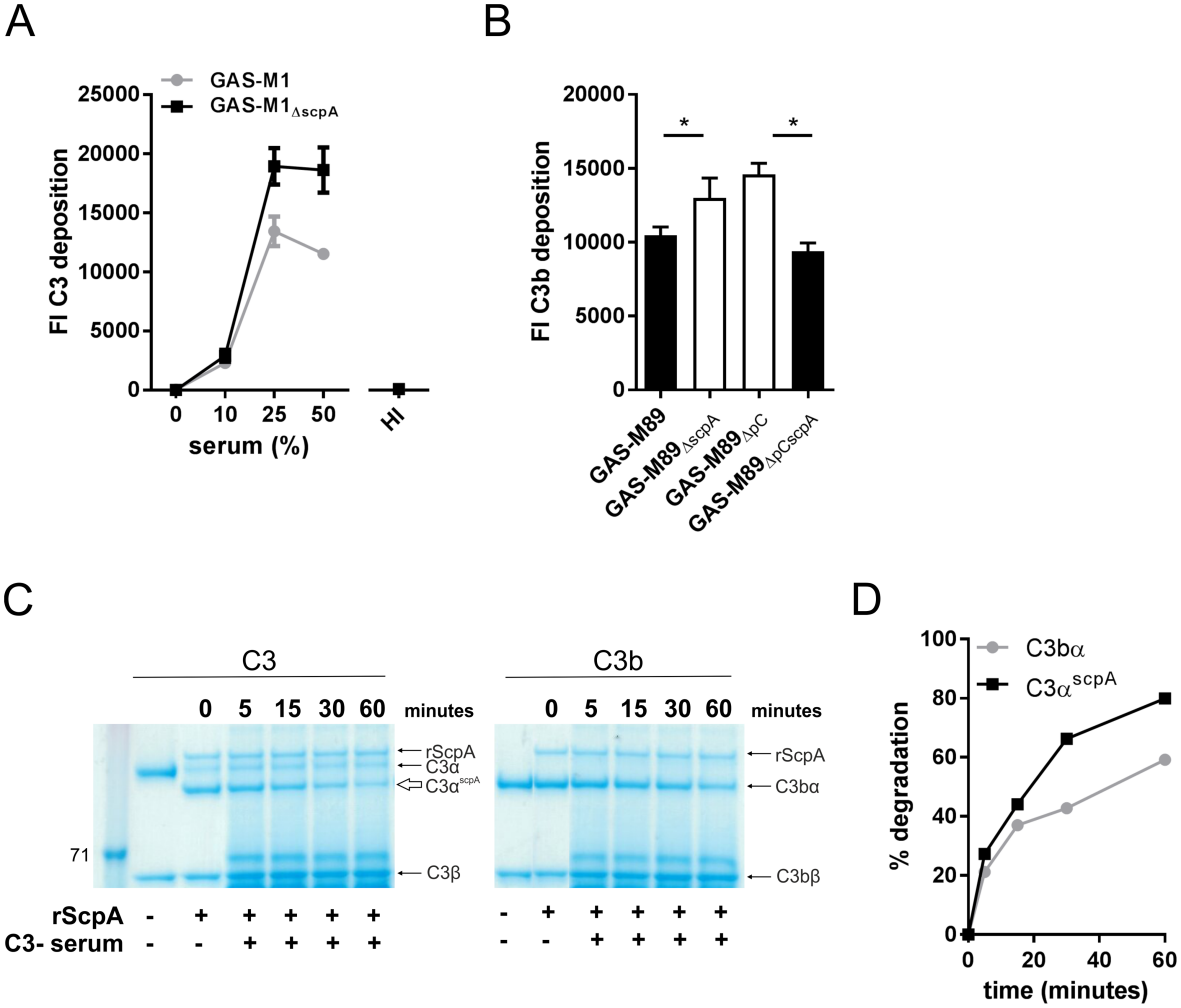
ScpA activity impairs C3 deposition on the GAS surface. A) C3 deposition on GAS-M1 and GAS-M1_ΔscpA_ surface was compared following incubation with 0% to 50% human serum. Deposition of C3 on the bacterial surface was quantified using FITC-conjugated anti-C3. Data represent mean +/- SD of 3 serum donors. C3 deposition was not seen when 50% heat-inactivated (HI) serum was used. B) GAS-M89 and isogenic ΔscpA strains were incubated with 50% human serum for 30 minutes, at 37°C. Deposition of C3 on the bacterial surface was quantified using FITC-conjugated anti-C3. Data represent mean +/- SD of 3–4 technical replicates produced from 3 serum donors. (One-tailed T-test * = p<0.05). In order to combine the percentage of C3-positive bacteria and the binding intensity, C3 deposition is presented as fluorescence index (FI), calculated as the proportion of positive bacteria expressed as a percentage multiplied by the gMFI [52]. C) Visualization of serum-mediated degradation of C3α chain. Degradation of physiological C3bα (right hand panel) and equivalent streptococcal ScpA cleavage product C3α^scpA^ (left hand panel) in serum was assessed over 60 minutes. Purified human C3 or C3b were incubated with rScpA for 16 hours, at 37°C. The relative degradation of resulting C3bα and C3α^scpA^ moieties by 1% C3-depleted serum was visualized by SDS-PAGE. Degradation of both C3α^scpA^ and C3bα was observed. D) The rate of degradation of physiological C3bα and the equivalent streptococcal cleavage product C3α^scpA^ was compared following quantification by densitometry and visualized as a line graph.

Cleavage of C3α-chain by ScpA results in the release of a longer C3b-like molecule (C3α^scpA^) compared with that produced by physiological C3 convertase (Fig 2). We hypothesized that this would impact on protein function, specifically C3 deposition on the bacterial surface, and thus went on to explore this in an experimental setting. Following incubation of live GAS with fresh human serum as a source of complement, the level of C3 deposition on the bacterial surface was quantified by flow cytometry. Initially, experiments were carried out using GAS-M1 and the isogenic GAS-M1_ΔscpA_ strain at a range of serum concentrations. Incubation of GAS with serum at a concentration of 25% or more resulted in the detection of significantly less C3 deposition on the surface of GAS-M1 compared with GAS-M1_ΔscpA_ (Fig 4A). Deposition of C3 fragments on GAS required active complement and was barely detectable when cognate heat-inactivated serum samples were used (Fig 4A), despite similar amounts of C3 being present in the heat-inactivated serum (S7 Fig). Serotype M1 GAS are known to inhibit complement deposition via recruitment of host fibrinogen to the bacterial surface by M1 protein [9]. In order to determine if our findings could be reproduced in GAS serotypes where M protein does not bind fibrinogen [27], we carried out C3 deposition experiments in isogenic M89 strains GAS-M89, GAS-M89_ΔscpA_, GAS-M89_ΔscpApC_ and GAS-M89_ΔpCscpA_ (Fig 4B). As observed for GAS-M1, loss of expression of ScpA resulted in enhanced C3 deposition on the bacterial surface, which could be reversed following complementation of GAS-M89_ΔscpA_ with ScpA expressed in *trans* (Fig 4B). These data suggest that ScpA contributes to inhibition of C3 deposition by multiple GAS serotypes and may allow escape from complement-mediated immunity.

The physiological relevance of the reduction in C3 deposition on the bacterial surface as a result of rScpA activity was further assessed by comparison with rAur. Both proteins led to comparable reduction in C3 deposition on the surface of GAS-M1_ΔscpA_ (S6B Fig). To better understand the mechanism by which C3 cleavage by GAS might contribute to a reduction in C3 deposition, we investigated the stability of the abnormally long C3α^scpA^ moiety, compared with regular C3bα-chain. C3α^scpA^ generated by rScpA cleavage was subject to rapid degradation by serum host factors when compared with physiological C3bα chain, indicating reduced stability (Fig 4C and 4D). Together, these data demonstrated that the C3α^scpA^ molecule generated by ScpA cleavage is functionally impaired in its ability to resist degradation by host serum factors and opsonize invading bacteria.

### Cleavage of C3 or C3a by ScpA significantly impairs host neutrophil function

**Fig 5:**
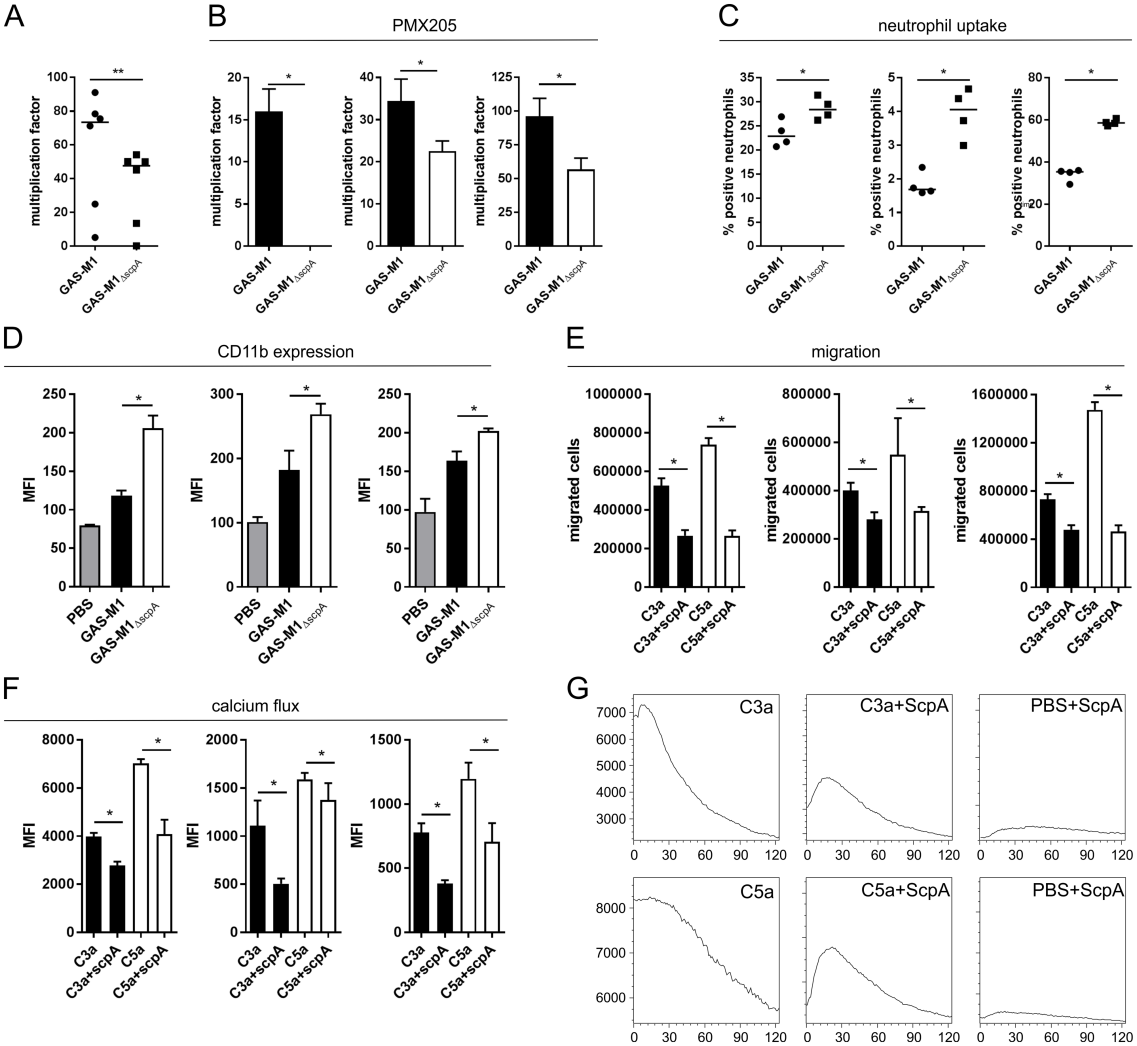
ScpA activity impairs the host neutrophil response to GAS. A) Survival of GAS-M1 and GAS-M1_ΔscpA_ in whole human blood was quantified by the classical Lancefield assay to determine resistance to neutrophil-mediated killing. Data represent six individual blood donors (each data point is mean of 4 technical replicates), line denotes median value (Mann Whitney U, ** = p<0.001). B) The role of C5a signaling on GAS survival in whole human blood was quantified by Lancefield assay. Whole blood was pre-treated with C5aR1 inhibitor PMX205 (1 μM) [28] prior to incubation with either GAS-M1 or GAS-M1Δ_scpA_. Each graph represents an independent donor (mean+/- SD 4 technical replicates, Mann Whitney U, * = p<0.05). C) Neutrophil-mediated uptake of fluorescent GAS-M1 and GAS-M1_ΔscpA_ was quantified following incubation with purified human neutrophils. Each graph represents percentage of neutrophils bearing fluorescent GAS from an independent donor (mean+/- SD 4 technical replicates, Mann Whitney U, * = p<0.05). D) Neutrophil activation was assessed by quantification of surface expression of CD11b on purified neutrophils following incubation with GAS-M1 or GAS-M1_ΔscpA_. Each graph represents an independent neutrophil donor (mean+/- SD 4 technical replicates, Mann Whitney U, * = p<0.05). E) Quantification of neutrophil migration along a chemokine gradient. Absolute number of purified human neutrophils migrating towards C3a or C5a +/- pre-treatment with rScpA was quantified following 30 minute incubation at 37°C. Each graph represents an independent neutrophil donor (4 technical replicates (mean +/- SD), Mann Whitney U, * = p<0.05). F-G) Neutrophil activation by C3a and C5a +/- pre-treatment with rScpA was quantified in a calcium mobilization assay. Calcium transients in fluo-4-AM labelled neutrophils were elicited by C3a or C5a, and calcium derived MFI was recorded on a FACSCalibur from 0–120 seconds. F) Overall MFI of all neutrophils at 120 seconds were compared. Each graph represents an independent neutrophil donor (4 technical replicates (mean +/- SD), Mann Whitney U, * = p<0.05). G) Calcium flux over 120 seconds was visualized following kinetic analysis. Data presented as a histogram of one donor, representative of 3 donors.

We hypothesized that the observed effects of C3 and C3a cleavage would affect bacterial clearance, as cleaved C3a might lack activity and C3b^scpA^ fragments would be less likely to promote enduring phagocytic uptake and killing by the host immune response. ScpA has previously been reported to promote streptococcal resistance to neutrophil-mediated opsonophagocytosis and survival in whole human blood, however the mechanism underlying this was not characterized [17]. While this phenotype could be attributed to loss of neutrophil activation by C5a, we also considered the possibility that the reduction in opsonization of GAS by C3 following cleavage by ScpA might impact on bacterial uptake and clearance as well. Consistent with a central role of ScpA in interference with phagocytic killing, bacterial survival in whole human blood, quantified by Lancefield Assay, was significantly reduced for isogenic mutants no longer expressing ScpA compared with wildtype parent M1 strains (Fig 5A). Importantly the same phenotype was also observed following blockade of CD88 (C5aR1) with the chemical inhibitor PMX205 (Fig 5B) [28], suggesting at least a partial C5-independent role for ScpA in inhibition of bacterial killing and growth in whole human blood. To confirm that neutrophil uptake was affected by ScpA, we compared the ability of purified human neutrophils to phagocytoze GAS-M1 and GAS-M1_ΔscpA_ following opsonization with human serum. Expression of ScpA enhanced bacterial resistance to neutrophil uptake (Fig 5C) and, while we believe the reduction in function of C3α^scpA^ compared with physiological C3bα contributes to this phenotype, we hypothesized that inactivation of C3a and C5a would also impact on neutrophil function.

C3a and C5a each display anaphylatoxin and chemoattractive properties, and as such play a key role in the activation and recruitment of neutrophils to the site of infection [25]. Using surface expression of CD11b (CR3) as a marker of activation we observed a significant reduction in neutrophil activation in the presence of ScpA following comparison of wildtype GAS-M1 with isogenic GAS-M1_ΔscpA_ (Fig 5D). This dampening of neutrophil activation suggested a significant loss of function of cleaved C3a and C5a, with potential far-reaching effects on neutrophil-mediated immunity. We hypothesized that neutrophil recruitment via C5a and C3a chemotactic gradients, and activation by calcium flux, would also be impaired. ScpA peptidase activity resulted in a significant reduction in neutrophil migration along both C3a and C5a gradients (Fig 5E). In line with these findings, the cytosolic calcium flux mediated by cleaved C3a^scpA^ and C5a^scpA^ fragments was significantly impaired compared with full-length molecules (Fig 5F and 5G). Importantly, exposure to recombinant ScpA alone did not induce a detectable increase in neutrophil intracellular calcium levels (Fig 5F and 5G). In conclusion, ScpA peptidase activity destroyed the complement chemotactic gradients required for migration of human neutrophils at the local site of infection and significantly impaired functional activation.

### C5a independent impact of ScpA *in vivo*

**Fig 6:**
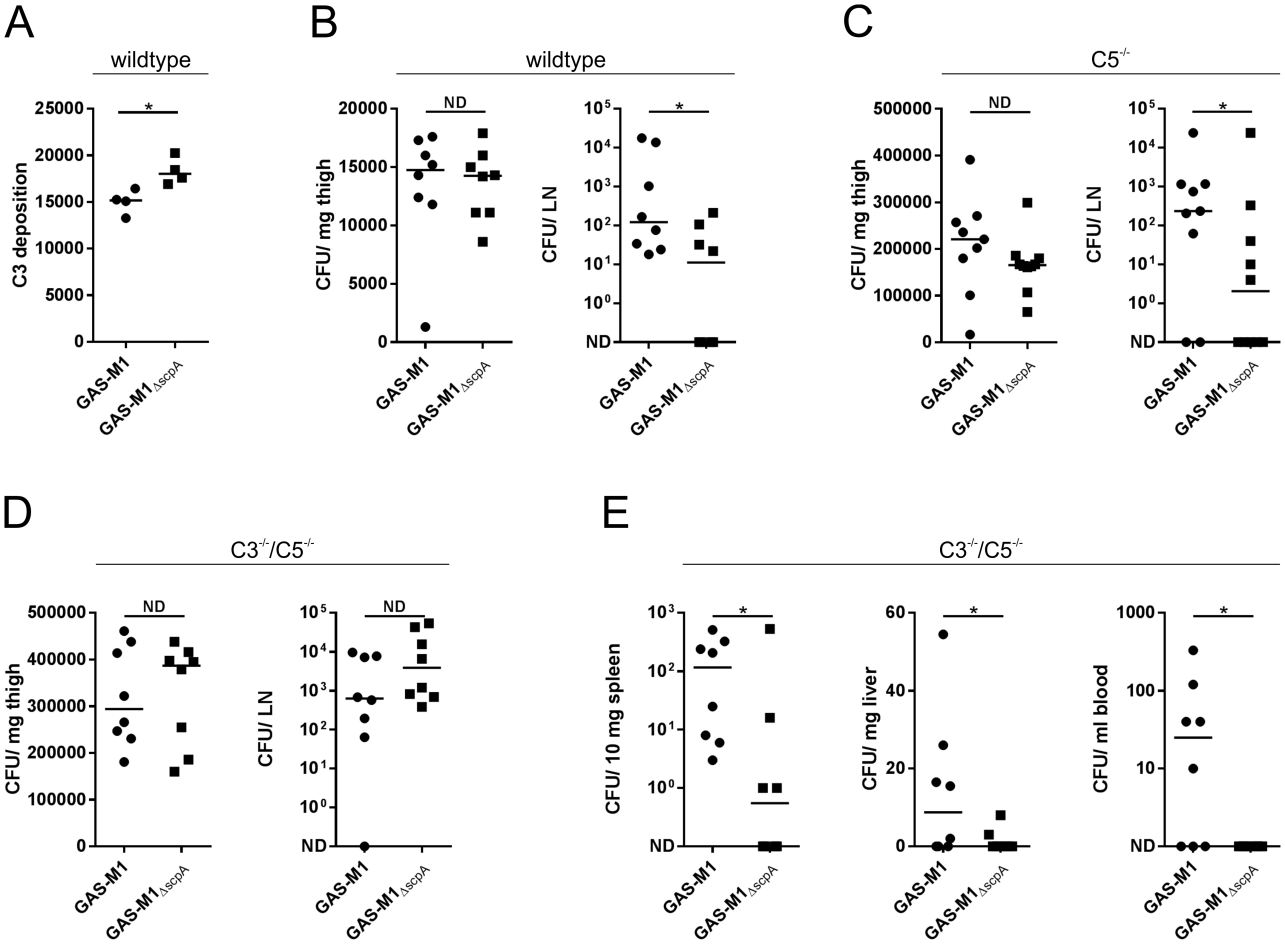
ScpA enhances streptococcal pathogenesis in wild type and complement deficient mice. A) C3 deposition on GAS-M1 and GAS-M1_ΔscpA_ surface was compared following incubation with 50% murine serum. Deposition of C3 on the bacterial surface was quantified using FITC-conjugated anti-mouse C3. Data are presented as fluorescence index (FI), calculated as the proportion of positive bacteria expressed as a percentage multiplied by the gMFI (line depicts median of four technical replicates Mann Whitney U, * = p<0.05). B-D) Characterization of GAS-M1 and GAS-M1_ΔscpA_ dissemination from the site of infection (thigh) to the draining inguinal lymph node (LN) in a murine model of soft tissue infection. Comparison of dissemination in B) wildtype C57BL/6 mice (n = 8/group), C) C5^-/-^ mice (C57BL/6 background) (n = 9/group) and D) C3^-/-^/C5^-/-^ mice (C57BL/6 background) (n = 8/group). Line depicts median value. (Mann Whitney U, No difference (ND) = p>0.05, * = p<0.05). E) Systemic spread of GAS-M1 and GAS-M1_ΔscpA_ in C3^-/-^/C5^-/-^mice. Quantitative culture of bacteria recovered from spleen, liver and blood of infected mice.

The structure of complement components is strongly conserved between species so we predicted that the peptidase activity of ScpA would be sufficient to mediate functional cleavage of murine (m) C3, C3a and C5a. Incubation of wildtype GAS-M1 or recombinant ScpA with mC3a and mC5a over 16 hours resulted in a clear reduction in size of both proteins when visualized by SDS-PAGE (S8A and S8B Fig respectively), similar to ScpA-mediated cleavage of human C3a and C5a (Fig 2). Genetic deletion of *scpA* in isogenic strain GAS-M1_ΔscpA_ removed the ability of GAS to cleave either murine C3a or C5a (S8A and S8B Fig). Importantly however, the rate of cleavage of C3a and C5a was significantly slower than observed for human counterparts (S8C and S8D Fig). A small reduction in C3 deposition on the surface of wildtype GAS-M1 compared with GAS-M1_ΔscpA_ was observed following incubation with fresh murine serum (Fig 6A), demonstrating that ScpA peptidase activity can influence murine C3-opsonization of GAS. We thus went on to determine whether ScpA might play a C5a-independent role in GAS disease outcome in a mouse model.

Soft tissue infections represent an important clinical manifestation of GAS, and can be readily modelled in mice to study the initiation and early events in clearance of infection that were previously implicated in ScpA activity [19]. Accordingly, we utilized a previously characterized model of soft tissue infection to ascertain the role of ScpA in the early stages of dissemination during infection [29], comparing pathogenesis of isogenic GAS-M1 and GAS-M1_ΔscpA_ in wildtype mice. Importantly we could detect ScpA expression at the site of infection in mice infected with GAS-M1, demonstrating the pathophysiological relevance of this virulence factor (S9 Fig). Local spread of GAS-M1 from the site of infection was significantly reduced following genetic deletion of *scpA* (Fig 6B). Three hours after intra-muscular infection, the bacterial burden at the site of infection was comparable between mice infected with either GAS-M1 strain, however dissemination to the locally draining inguinal lymph node was significantly reduced in mice infected with GAS-M1_ΔscpA_ (Fig 6B). At this early timepoint, systemic spread of GAS-M1 was not detectable. By 24 hours however, any difference between strains was no longer detectable, despite systemic spread to spleen, liver and blood (S10 Fig). To investigate these early pathogenic events, histological sections of thigh were examined from C57Bl/6 mice infected with GAS-M1 or GAS-M1_ΔscpA_ (n = 4/group). At this early, 3 hour time-point, bacteria could only be detected in some sections (GAS-M1: 3/4, M1_ΔscpA_: 1/4) (S11 Fig); any neutrophil infiltrate was scant and, under high magnification, appeared necrotic in mice infected by either GAS-M1 strain (S11B Fig). Taken together the quantitative data suggested that the early clearance and local containment of infecting GAS was significantly impaired by ScpA, however, we were unable to detect a specific effect on neutrophil recruitment at this time point histologically, despite our results with human cells.

In order to ascertain whether the observed effects of ScpA were independent of any effect on C5 or C5a cleavage, the same experiment was carried out in age and weight-matched C5^-/-^ mice (Fig 6C). Following infection via the intra-muscular route, dissemination of GAS-M1 and GAS-M1_ΔscpA_ was determined by quantitative culture. While no difference was observed at the site of infection, we again found that considerably fewer bacteria were recovered from the draining inguinal lymph node of mice infected with GAS-M1_ΔscpA_. This suggested that ScpA might be affecting local GAS spread to lymph nodes in a C5a independent manner.

To determine whether these results were indeed due to C3-ase activity of ScpA, we carried out the same experiment in age and weight-matched C3^-/-^/C5^-/-^ knockout mice that are essentially complement deficient (Fig 6D and 6E). No difference was observed between GAS-M1 and GAS-M1_ΔscpA_ infected groups in clearance from the site of infection to the locally draining lymph node (Fig 6D), suggesting that C3-mediated clearance may be more important than C5 in the lymphatic system. However, surprisingly, expression of ScpA significantly enhanced GAS systemic dissemination to blood, spleen and liver (Fig 6E). These unexpected results pointed to a complement-independent role for ScpA in systemic bacterial clearance in settings where there is no C3 and C5.

**Fig 7:**
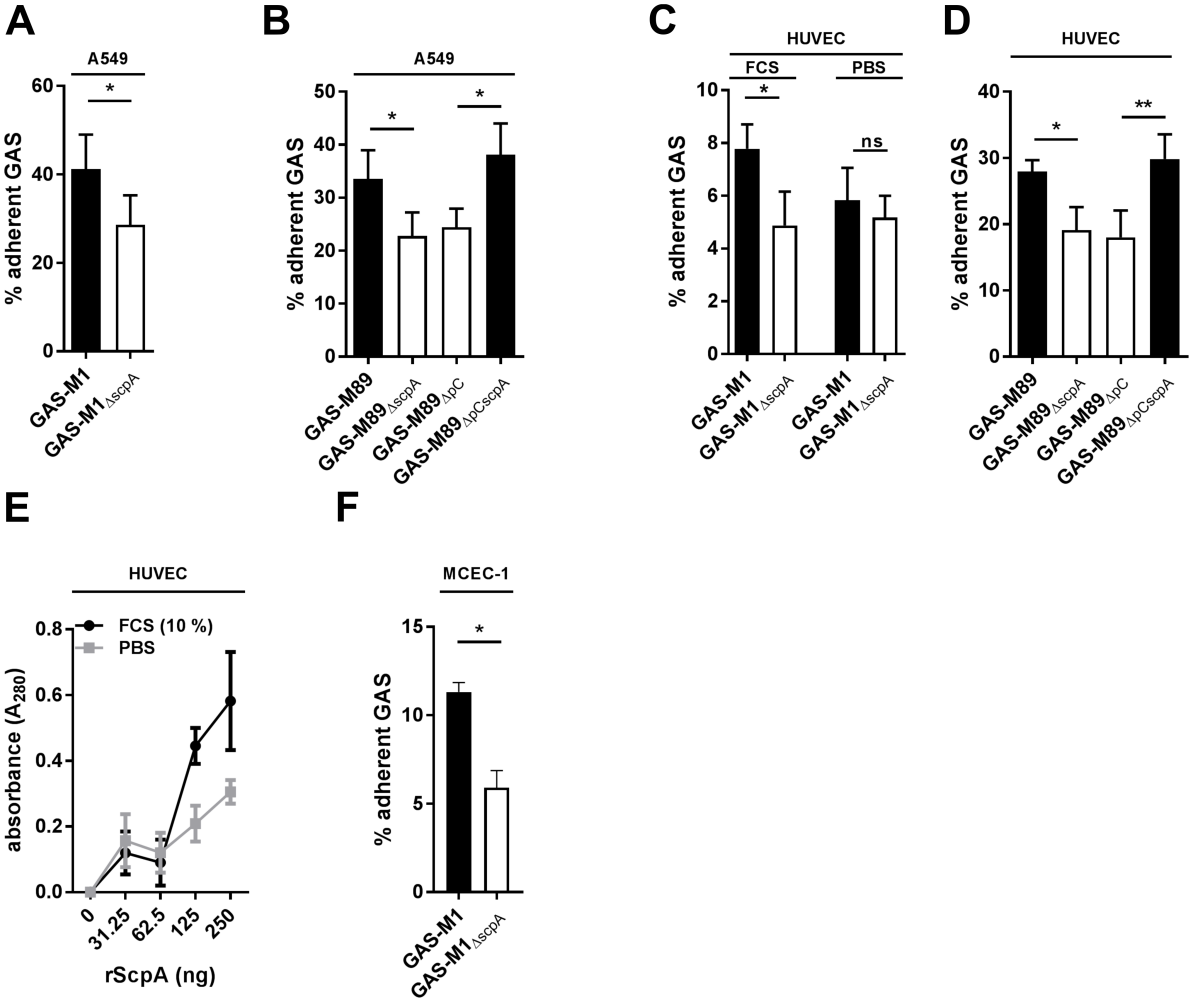
ScpA requires serum factors to mediate GAS adhesion to epithelial and endothelial cells. A+B) Adhesion of A) GAS-M1 and B) GAS-M89 and isogenic ΔscpA strains to A549 epithelial cells was compared by quantitative culture (30 min incubation). Data represent mean+/-SD of 3 experimental replicates (T-test * < 0.05). C+D) Adhesion of C) GAS-M1 and D) GAS-M89 and isogenic ΔscpA strains to primary human HUVEC was compared by quantitative culture (30 min incubation). Data represent mean+/-SD of 3 experimental replicates (T-test * < 0.05, ** < 0.001). E) Adherence of rScpA protein to paraformaldehyde-fixed human HUVEC. Bound rScpA was quantified following incubation with anti-ScpA mouse serum and goat-anti mouse HRP antibody and detection with TMB. Adhesion was compared in the presence and absence of 10% FCS. Data represent mean+/-SD of 3 experimental replicates (T-test * < 0.05). F) Adhesion of GAS-M1 and GAS-M1_ΔscpA_ to the murine endothelial cell line MCEC-1 was compared by quantitative culture (30 min incubation). Data represent mean+/-SD of 3 experimental replicates (T-test * < 0.05).

ScpA has been implicated as an adhesin for both GAS and GBS, however an adhesin effect in live bacteria interacting with live host cells has not been directly demonstrated [30, 31, 32]. We hypothesized that the enhanced systemic dissemination of GAS-M1 compared with GAS-M1_ΔScpA_ in the absence of complement might be the result of adhesion to and subsequent invasion through endothelial tissue. However, in natural infection, interactions with epithelial cells may be important in initial colonization events. In order to test this hypothesis we went on to characterize the ability of the A549 human lung epithelial cell line and primary human umbilical vein endothelial cells (HUVEC) to support attachment of GAS-M1 and GAS-M89 (Fig 7A+B and 7C+D respectively). After demonstrating that both GAS serotypes could adhere to these cell monolayers, we went on to compare and quantify adherence of isogenic ScpA positive and negative strains. Expression of ScpA enhanced attachment of GAS-M1 and GAS-M89 to A549 (Fig 7A and 7B) and HUVEC (Fig 7C and 7D), but had no effect on passive internalization at this 30 minute time-point (S12 Fig). rScpA protein was also able to bind to fixed HUVEC in a dose-dependent manner (Fig 7E). Interestingly, fixed HUVEC monolayers were able to support greater binding of rScpA in the presence of serum (10% FCS) (Fig 7E), an effect which was also seen for attachment of GAS-M1, but not GAS-M1_ΔScpA_ to live HUVEC monolayers (Fig 7C). Expression of ScpA was also sufficient to promote GAS-M1 adhesion to murine cardiac endothelial cells (MCEC-1) (Fig 7F), suggesting that this phenotype could explain the enhanced virulence displayed by GAS-M1 in the murine model (Fig 6). These data support a role for ScpA as an adhesin for attachment of live GAS to epithelial and endothelial cells, which may contribute to the invasive potential of this bacterium *in vivo*.

**Fig 8:**
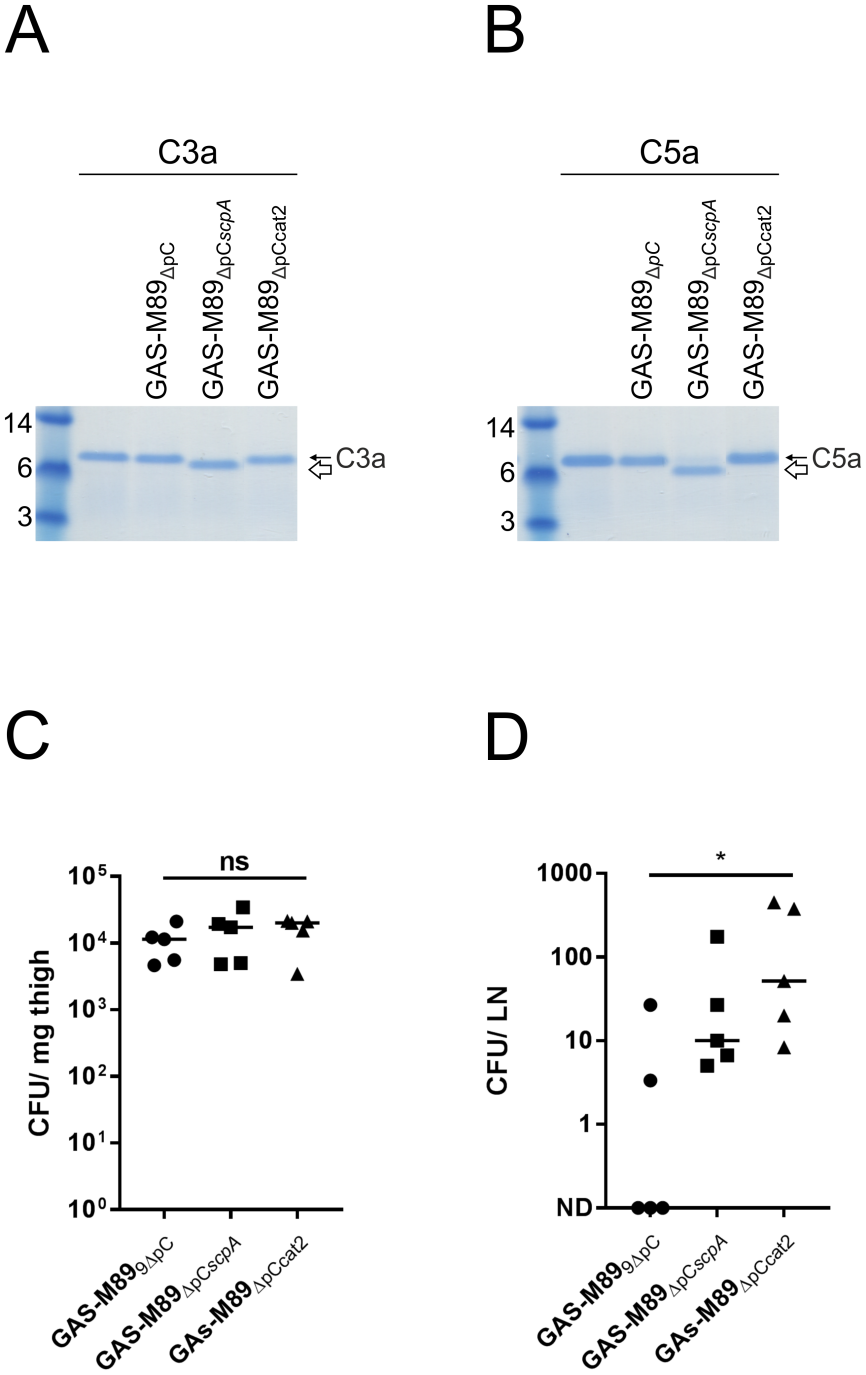
Catalytic activity of ScpA is not required for pathogenesis *in vivo*. A+B) Cleavage of human complement factors A) C3a and B) C5a by whole cell pellets (4x10^6^ cfu) of GAS-M89_ΔpC_, GAS-M89_ΔpCscpA_ and GAS-M89_ΔpCcat2_. Only strain GAS-M89_ΔpCscpA_, expressing catalytically active ScpA was able to cleave either complement factor. Cleaved moieties (C3a^scpA^ and C5a^scpA^) are indicated by a white arrow in each panel. C+D) Characterization of GAS-M89_ΔpC_, GAS-M89_ΔpCscpA_ and GAS-M89_ΔpCcat2_ dissemination in a murine model of soft tissue infection in wildtype CD57/BL6 mice (n = 5/group). Quantitative culture of tissue homogenates (C) from the site of infection (thigh) to (D) the draining inguinal lymph node (LN). Line depicts median value. (Mann Whitney U, No difference (ns) = p>0.05, * = p<0.05).

In order to better define the role of ScpA during infection, we generated strain GAS-M89_ΔpCcat2_ which over-expresses enzymatically inactive ScpA due to a mutation in the second amino acid of the catalytic triad His_193_ to alanine [16]. The catalytically inactive ScpA included an additional SNP not predicted to affect structure or expression. We went on to confirm that this mutant was no longer able to cleave C3a (Fig 8A) or C5a (Fig 8B), in contrast to plasmid-transformed GAS-M89_ΔpCscpA_ that over-expressed intact ScpA, consistent with previously reported ScpA structure function studies [16]. To ascertain whether the observed ScpA-enhanced dissemination of GAS (Fig 6) was dependent on catalytic activity, we compared the virulence of strains GAS-M89_ΔpC_, GAS-M89_ΔpCscpA_ and GAS-M89_ΔpCcat2_ during soft-tissue infections in age and weight-matched C57BL/6 mice.

Following infection via the intra-muscular route, bacterial dissemination was determined by quantitative culture. As we found previously for GAS-M1 and GAS-M1_ΔScpA_ (Fig 6B), no difference was observed in bacterial counts recovered from the site of infection (Fig 8C). Intriguingly, greater numbers of GAS were recovered from the draining inguinal lymph node of mice infected with GAS expressing ScpA, regardless of whether the ScpA was catalytically active (Fig 8D). This demonstrated that the properties of ScpA that were critical for pathogenesis in the mouse model were independent of any enzymatic activity, pointing to a potentially important role for adhesion in disease progression through interactions with endothelial or epithelial cells.

It was not possible to deduce the relative contribution of ScpA adhesion to pathogenesis in a human system, albeit that ScpA conferred adhesion to human and murine cells. The discrepancy in rates of ScpA-mediated cleavage of human and murine complement factors (Fig 3, S8A and S8B Fig) and the smaller reduction in murine C3 (Fig 6A) compared with human C3 (Fig 4A) deposited on the GAS-M1 surface raised the possibility that, in human disease, the impact of complement cleavage may be of greater importance than adhesion. Our study highlights the multi-functional mechanisms by which ScpA is able to promote bacterial invasion, through a combination of factors including adhesion and active inactivation of the innate immune response.

## Discussion

Streptococcal C5a peptidase is expressed by a variety of streptococcal species. It thus is believed to play a pivotal role in the success of the streptococci, however to date its role in immune evasion has largely been attributed to its ability to cleave C5a. In this work we have identified multifunctional effects of ScpA which include rapid cleavage and inactivation of the anaphylatoxin C3a as well as cleavage of the central complement protein C3. In addition, we have demonstrated complement-independent activity of ScpA as an adhesin, that may contribute to systemic spread during infection *in vivo*. The complement cascade plays a crucial role in the innate immune response to invading bacteria and as such represents a key target for pathogen immune evasion [1,3,4,10,22,33,34]. The inactivation of both anaphylatoxins C3a and C5a as well as the central complement component C3 we report here has the potential not only to reduce inflammation and cellular activation and recruitment at the site of infection, but also to dampen amplification of the complement system in response to infection, a phenomenon that has not previously been reported for the Group A *Streptococci*.

ScpA is expressed by all GAS serotypes, and homologous enzymes are encoded by multiple other Lancefield groups [35–38]. Conservation of enzyme function both within and between species points to a clear functional evolutionary pressure to systematically disrupt neutrophil responses [36], despite ScpA being a primary target for opsonizing or neutralizing antibodies produced during infection [38–43]. ScpA is a member of the subtilisin-like family of serine proteases which are reported to cleave multiple enzymatic substrates, and, while the streptococcal protease SpyCEP cleaves numerous ELR+ CXC chemokines [24], C5a was hitherto the only reported substrate for ScpA.

Our investigation into C3 cleavage by streptococci began following the identification of a putative “C3 degrading protease” (CppA) [21] although no such activity could be ascribed to CppA. Initial analyses established that groups A, C and G streptococci were, however, capable of specifically cleaving full-length C3, a function that has not previously been reported for these species. Furthermore, cleavage activity could be inhibited by the serine protease inhibitor Pefabloc, which led us to hypothesize that ScpA could be mediating C3 cleavage, plausible given the structural similarity between C5a and C3a [25]. The cleavage of C3 that we observe begins within 30 minutes incubation with rScpA protein. Whilst we believe this to be physiologically relevant during infection, it may be that large numbers of bacteria, such as at the site of infection, are required for ScpA C3-ase activity to play a significant role in pathogenesis.

The C3 molecule is an obvious target for pathogen immune evasion, and, as such, other bacterial species also express inactivating enzymes. Cleavage of C3 generating a longer C3b^scpA^ and a shorter C3a^scpA^ molecule by GAS reproduces the mechanism described for the *N*. *meningitidis* C3-ase NalP [26]. The reported C3-cleaving enzymes of other Gram positive pathogens, *Staphylococcus aureus* (aureolysin) [12] and *Enterococcus faecalis* (GelE) [44], which both cleave 2 amino acids upstream of the physiological C3 convertase site, generate a smaller C3b and longer C3a moiety, in contrast to our findings for ScpA. Cleavage of C3a is thus a function uniquely shared by ScpA and NalP [26], however any effect of such cleavage on C3a activity has not previously been reported.

The GAS cysteine protease SpeB has previously been reported to degrade C3b in human serum, where ScpA could not [11]. In our study, we examined full-length C3, which we demonstrate could be cleaved by ScpA, but not SpeB. This suggests that the various C3-ase functions of these enzymes are distinct, yet may act synergistically to overcome the host innate immune response. C3 is present in human serum at approximately 1 mg/ml, therefore multiple strategies for effective inactivation may be necessary to overcome this highly abundant molecule. Although ScpA is known to be regulated by Mga, our preliminary experiments using available isogenic strains deficient in functional CovR/S or RocA (Regulator of Cov), showed that ScpA was also a member of the CovR regulon (S13 Fig). Both SpeB and ScpA are regulated by CovR/S, however they display reciprocal expression profiles. It is therefore possible that each protein acts at a different phase of infection to modulate the host immune response. SpeB expression is significantly reduced in naturally occurring CovR/S mutants, which are frequently associated with invasive infection. As such, ScpA, which is expressed by invasive and non-invasive disease isolates, may represent a common mechanism of action shared amongst GAS isolates.

The significance of ScpA as a standalone virulence factor was assessed by the generation of isogenic mutants in two GAS serotypes. Loss of function had a significant effect on C3 deposition and stability, and on neutrophil activation by the anaphylatoxins C3a and C5a. Indeed, cleavage of these two proteins reduced neutrophil recruitment along chemotactic gradients and impaired activation, specifically upregulation of CD11b and induction of intracellular calcium release which normally play an important role in pathogen phagocytosis. Fibrinogen binding by the M1 protein is known to have a major influence on GAS-M1 clearance and may render other virulence factors redundant [9]. Nonetheless, ScpA had a measurable effect on C3 deposition on GAS-M89 (where fibrinogen binding is not thought to be dominant) [27] and also in whole blood assays that contain human fibrinogen, both in the presence and absence of a C5aR1 antagonist, suggesting that the C5-independent activities we describe are detectable even in the presence of fibrinogen.

In order to determine the functional relevance of the novel C3 and C3a cleavage activity, *in vivo* experiments were initially performed in both wildtype and C5^-/-^mice. Interestingly, similar results were obtained in both genetic backgrounds, suggesting that ScpA plays a role in streptococcal pathogenesis that is independent of C5a cleavage. Importantly, cleavage of C3 not only impairs C3a and C3b function, but also reduces generation of C3b and C5a. Generation of C3b via the alternative pathway is amplified by activity of the C3 convertase, which is comprised of multiple subunits including C3b. Reduction in activity of the alternative pathway C3 convertase will subsequently reduce activation of terminal complement, one output of which is functional C5a. Moreover, C3b is essential for formation of the C5 convertase, and, as such, inactivation of C3 will significantly impair C5a generation and reduce the activity of the entire complement cascade [13]. Thus, in combination with other pan-streptococcal cell envelope serine proteases such as SpyCEP, which cleaves the potent chemoattractant IL-8/CXCL8 and other ELR+ CXC chemokines [23], GAS is capable of systematic inhibition of neutrophil migration and activation, possibly explaining the dearth of functional neutrophils recruited to the site of infection during severe clinical invasive disease.

To validate a clear role for the C3-ase activity of ScpA *in vivo*, utilizing the same soft-tissue infection model, we compared the pathogenesis of GAS-M1 and GAS-M1_ΔscpA_ in complement deficient (C3^-/-^/C5^-/-^) mice. The difference in clearance of bacteria from the site of infection to the locally draining inguinal lymph node observed in both wildtype and C5 deficient mice was diminished in C3^-/-^/C5^-/-^ mice, suggesting that cleavage of C3 may be responsible for this phenotype. However, systemic dissemination via the blood was significantly impaired in the absence of ScpA, suggesting an additional complement-independent role for this protein in augmenting systemic spread.

Using isogenic GAS strains and recombinant protein, we were able to show that ScpA is a potent adhesin, important for bacterial attachment to both human epithelial and endothelial cells. We went on to demonstrate that ScpA could also mediate attachment of GAS-M1 to murine endothelial cells, providing support for the hypothesis that adhesion was playing a key role in GAS pathogenesis in our *in vivo* model. Using bacteria expressing ScpA constructs that differed only in catalytic activity, we found that the enzymatic activity of ScpA was not necessary for pathogenicity in a murine model of soft tissue infection, noting the presence of an additional mutation in the enzymatically inactive construct that is not predicted to affect structure or expression. While extrapolating the relevance of our findings to human infection is beyond the scope of this study, the data strongly implicate complement-independent functions of ScpA in streptococcal virulence and disease progression in human infection. We speculate that the failure to control GAS infection in humans can be attributed in part to the complement-independent ability of ScpA to act as an adhesin for endothelial and other cell types, which may play a key role in infection where complement is absent.

Here we have identified complement factors C3 and C3a as two novel substrates of the streptococcal enzyme C5a peptidase and demonstrated that the cleavage of these factors has significant functional effects on the host immune system, most explicitly on human neutrophil activation and function. We have also characterized the role of ScpA as an adhesin, important for attachment to epithelial and endothelial cells. The multiple functions of ScpA described demonstrate its fundamental role in streptococcal disease, and underline the importance of assigning novel functions to known virulence factors as a means to provide new insight into disease progression and pathogenesis.

## Methods

### Ethics statement

Human blood from anonymized consenting healthy donors was obtained from an approved subcollection of the Imperial College NHS Trust Tissue Bank (ICHTB reference R12023). *In vivo* experiments were performed in accordance with the Animals (Scientific Procedures) Act 1986, and were approved by the Imperial College Ethical Review Process (ERP) panel and the UK Home Office (Project Licence 70/7379).

### Bacterial strains

The strains used for molecular manipulation were invasive disease isolates GAS-M1 (H598) [14] and GAS-M89 (H293) [45]. All strains used are listed in Table 1. GAS were cultured on Columbia horse blood agar plates (OXOID) Todd-Hewitt (TH) agar or in TH broth (OXOID) at 37°C, 5% CO_2_ for 16 hours. *E*. *coli* XL-10 gold (Stratagene), TOP10 (ThermoFisher Scientific) BL21(DE3) (ThermoFisher Scientific) were grown in LB broth. Growth media were supplemented with antibiotics where appropriate at the following concentrations; *E*. *coli* erythromycin 500 μg/ml, kanamycin 50 μg/ml, ampicillin 100 μg/ml; GAS erythromycin 1 μg/ml, kanamycin 400 μg/ml.

**Table 1:** Strains used in this study.

Strain	Gene alteration	Reference/source
GAS-M1	Identical to H598	[14]
GAS-M1_ΔscpA_	ScpA mutant	This study
GAS-M89	Identical to H293	[45]
GAS-M89_ΔscpA_	ScpA mutant	This study
GAS-M89_ΔpC_	Above + pOri	This study
GAS-M89_ΔpCscpA_	Above + pOri_scpA_	This study
GAS-M89_ΔpCcat2_	Above + pOri_scpAcat2_	This study
GAS-M49	Identical to CS101	[18]
GAS-M49_ΔspeB_	*speB* disruption mutant	[46]
GAS-M81	Identical to H292	[24]
GAS-M81_ΔspyCEP_	*spyCEP* disruption mutant (H575)	[24]
*S*. *equi*	Identical to *Se*4047	[47]
*S*. *zoo*	Identical to *Sz*H70	[47]
GGS	H819	This study
M1 CovR+	Identical to H584	[14]
M1 CovR-	Animal-passaged H584 (CovR T65P mutant)	Kind gift from L. Lamb
M75 CovR/S+	Identical to H347	[48]
M75 CovR/S-	CovR/S disruption mutant (H494)	[48]
M18 RocA-	Identical to H566	[21]
M18 RocA+	H566_rocAM89_	[21]
M89 RocA-	GAS-M89_rocAM18_	[21]

### Polymerase chain reaction and DNA sequencing

Genomic DNA was extracted from GAS cultures grown to late logarithmic growth phase (OD_600_ 0.7–0.9) as described previously [21]. PCR was carried out using a MyCycler (Bio-Rad) thermal cycler with Bio-X-Act proof reading Taq (Bioline). Automated-fluorescent sequencing of PCR products and plasmids was performed by the MRC CSC Core Genomics Laboratory, Hammersmith Hospital.

### Construction of *scpA* disruption mutant

Deletion of *scpA* in GAS-M1 from nucleotide 333–1777 and GAS-M89 from nucleotide 333–2017, resulting in removal of the characterized catalytic domain (S3 Fig). A 500 bp fragment of the *scpA* gene was amplified (GAS-M1: forward primer: 5’- GGAATTCGCTTTAATAATCGTCCATGG -3’, reverse primer: 5’- GGGGTACCCCTCAAGCAAGGTTCACCTG -3’; GAS-M89: forward primer: 5’- GGAATTCTTCGATATCCTCTATGTTTTC -3’, reverse primer: 5’- GGGGTACCGAGTTGTATTACCAAGCAAC -3’) incorporating EcoRI and KpnI restriction sites into the 5’ and 3’ ends respectively, and cloned into the suicide vector pUCMUT to produce pUCMUT_GAS-M1_ and pUCMUT_GAS-M89_. A 3’ 500 bp fragment of the *scpA* gene was amplified (GAS-M1: forward primer: 5’- ACGCGTCGACGACCTGAGAAGGGTCGTTC -3’, reverse primer: 5’- AACTGCAGCTGTATCATATGCAAATAACC -3’; GAS-M89: forward primer: 5’- ACGCGTCGACCTGAGAAGGGTCGTTCAAATC -3’, reverse primer: 5’- AACTGCAGCTGTATCATATGCAAATAACC -3’) incorporating PstI and SalI restriction sites into the 5’ and 3’ ends respectively, and cloned into PstI/SalI digested pUCMUT_GAS-M1_ and pUCMUT_GAS-M89_ respectively. The constructs were introduced into GAS-M1 and GAS-M89 by electroporation and incorporated chromosomally by homologous recombination as described previously [21]. PCR analysis demonstrated that only a single recombination event had occurred. For complementation *scpA* was cloned into replicative vector pOri23 (scpApOri F: 5’- CCGGATCCCATCAGGAAAGGACGACACATTGC-3’, scpApOri R: 5’- CCGGATCCGATCAGTTGTACTAATCTTCAGTGC-3’) incorporating in BamHI sites at both 5’ and 3’ termini. pOri_scpA_ was propagated in One Shot^®^ TOP10 Chemically Competent E. coli (Invitrogen) and transformed into electrocompetent GAS as described previously [21]. For generation of a complementation vector expressing catalytically inactive ScpA, the second amino acid of the catalytic triad His_193_, was mutated to alanine in vector pOri_ScpA_ by site directed mutagenesis (QuikChange XL-II Site-Directed Mutagenesis Kit, Stratagene) (forward primer: 5′- GCTGTCGATCAAGAGGCCGGCACACACGTGTC -3′, reverse primer: 5′- GACACGTGTGTGCCGGCCTCTTGATCGACAGC -3′) to produce vector pOri_scpAcat2_. Vector pOri_scpAcat2_ was sequenced and the desired mutation at residue 193 (His-Ala) confirmed. An additional non-synonymous SNP at position 243 (Ala to Thr) was not deemed to be relevant as it was not within any domains predicted to mediate adhesion. Full-length ScpA protein of size ~120 kDa was confirmed by western blotting and equivalent to that produced by pOri_scpA_. The vector was transformed into electrocompetent GAS-M89_ΔscpA_ as described previously [21].

### Recombinant protein expression

Recombinant proteins were produced using the following primer sequences (CppA: cppA_pET-F: 5’- CCGGATCCAATGACTTTAATGGAAAATATTAC -3’ cppA_pET-R: 5’ CCGGATCCTTCATTTCGTAAACCATACTTC 3’, HVR: emm_pET-F: 5’- CCGGATCCAGGTTTTGCGAATCAAACAGAGGTTAAG -3’, emm_pET-R: 5’- CCGGATCCTAGCTCTCTTAAAATCTCTTCCTGCAACTTCC -3’, Aur: aur_pET-F: 5’- CGGGATCCGATTGATTCAAAAAATAAACC -3’ aur_pET-R: 5’- CGGGATCCTTACTCCACGCCTACTTCATTC) and the pET-19b overexpression system as previously described [49]. rCppA, rEmm 1.0 hypervariable region (HVR), rAur and the previously described rScpA fragment [49], truncated at amino acid 720 to prevent pro-peptide removal, were purified using the Ni-NTA purification system (Invitrogen) according to the manufacturer's instructions. Purified proteins were buffer-exchanged into PBS. Full-length rScpA was expressed following amplification from gDNA (scpA_pET-F: 5’- CCGGATCCAACCAAAACCCCACAAACTC-3’, scpA_pET-R: 5’- CCGGATCCTAGAGTGGCCCTCCAATAGC-3’), and purified from BL21 cell lysates by size exclusion chromatography using a 1 × 50 cm Econo-Column^®^ (Bio-Rad) packed with Sephadex G-100 (Pharmacia). PBS fractions containing ScpA were identified by SDS-PAGE, pooled and concentrated using a 30,000 MWCO spin column (Vivaspin, GE Healthcare).

### Cleavage of complement factors and visualization by SDS-PAGE

16 hour timepoint: Recombinant ScpA (100 ng) or stationary phase GAS pellets (4x10^6^ cfu) washed twice in sterile PBS and concentrated 4x, were incubated with C3 (1 μg) (MD Millipore), C3a (0.5 μg) (R&D) or C5a (0.5 μg) (R&D) in THB in a final volume 10 μl. Protease inhibitor kit (Roche) was used, and each inhibitor used at concentrations described previously [23]. All samples were incubated for 16 hours, 37°C, 5% CO_2_. Pellets were centrifuged and resulting supernatants analyzed. Timecourse: 0.5 μM rScpA or rAur was incubated with C3 (0.5 μM) (MD Millipore), C3a (5 mM) (R&D) or C5a (5mM) (R&D) in THB or HEPES++ buffer (20 mM HEPES, 140 mM NaCl, 5 mM CaCl_2_, 2.5 mM MgCl_2_). Samples were fractionated on 3–8% Tris-acetate gels (C3 and C3b) or 4–12% Bis-Tris plus gels (C3a and C5a) (ThermoFisher Scientific) and stained with Instant Blue (Expedeon).

### N-terminal sequencing

To determine the ScpA cleavage site, 8 μg purified C3 (EMD Millipore) were cleaved with recombinant ScpA in THB for 16 hours, 37°C. Following cleavage, fragments were subjected to SDS-PAGE and transferred onto Hybond-LFP (Amersham). Membranes were stained with Amido Black (Sigma) and destained (25% v/v isopropanol, 10% v/v acetic acid). Cleaved C3α^scpA^ fragment was excised and subjected to sequencing by Edman degradation (AltaBioscience, Birmingham UK).

### Antibodies

Primary antibodies: For western blot, chicken anti-C3 (Abcam ab14232) (1:10,000), goat anti-his (Invitrogen) (1:50,000), rabbit anti-SIC [14] and rabbit anti-SpyCEP [24] serum (1:1000). Mouse antisera against Emm 1.0 HVR and the truncated ScpA molecule were raised by vaccination as described previously [49] and used at 1:1000 dilution. Secondary antibodies used were HRP-conjugated goat anti-chicken IgY, anti-mouse IgG and anti-rabbit IgG (Abcam) (all used at 1:80,000). For C3 deposition, FITC conjugated goat anti-human C3 and goat anti-mouse C3 antibodies (MP Biomedicals) were used (1:300).

### Preparation of streptococcal cell wall extracts/supernatants and immunoblotting

Cell wall extracts and bacterial supernatants were prepared as described previously [50] with minor modifications. Briefly, stationary phase cultures were pelleted and supernatants were 0.2 μm filtered. Cell pellets were incubated in cell wall extraction buffer (30% raffinose, 1 μg/ml lysozyme, 10 mM Tris-HCl (pH 8)) at 37°C, 3 hours and cell wall fraction was isolated by centrifugation. Raffinose was removed following dialysis into PBS using Slide-A-lyzer cassettes (ThermoFisher Scientific), and concentrated to 100 μg/ml. 1 μg cell wall extract was loaded for each sample to allow direct comparison between strains. Samples were separated on 4–12% Bis-Tris plus gels (ThermoFisher Scientific) and transferred onto Hybond-LFP membrane (Amersham). Membranes were blocked (5% skimmed milk powder (Sigma), 0.05% Tween (Sigma) and probed and detected with relevant primary and secondary antibodies. Membranes were developed using ECL Advance western blotting detection system (GE Healthcare).

### Degradation of C3bα^scpA^ fragment

Following cleavage of C3 with recombinant ScpA, the resulting fragments, or purified C3b (MD Millipore) were incubated with 1% C3-depleted human serum (MD Millpore) for 60 minutes. Samples were taken at 5, 10, 15, 30 and 60 minute timepoints. The reaction was stopped following addition of LDS sample buffer (ThermoFisher Scientific) and 0.1mM DTT, and degradation visualized by western blot using chicken anti-C3 (Abcam).

### Capsule quantification

GAS strains were cultured in THB and capsule quantified as described previously [21] using the hyaluronan DuoSet (R&D).

### C3 deposition assay

Stationary phase cultures of GAS (1x10^7^ cfu) were washed and resuspended in fresh human serum (0–50%) or mouse serum (50%) and incubated for 30 minutes, 37°C with end-over-end rotation. Control samples were incubated with fresh heat-inactivated human serum and human or mouse C3-depleted serum (EMD Millipore). Cells were stained with FITC-conjugated anti-C3 antibody (MP Biomedicals) for 30 minutes on ice [51] and analyzed using a FACSCalibur cell analyzer (BD Biosciences) and FlowJo Software (Treestar, OR). In order to combine the percentage of C3-positive bacteria and the C3-binding intensity, the results are presented as fluorescence index (FI), which is calculated as the proportion of positive bacteria expressed as a percentage multiplied by the gMFI) [52].

### Human whole blood phagocytosis assay

Lancefield assays were performed to assess GAS resistance to human phagocytic killing. GAS were cultured to OD_600_ 0.15 in THB, and diluted in sterile PBS. Approximately 50 GAS cfu were inoculated into heparinized whole human blood obtained from healthy volunteers, and incubated for 3 hours at 37°C with end-over-end rotation. Quantitative culture at this time point was carried out to determine the number of surviving bacteria. To ascertain the role of ScpA-mediated C5a cleavage, C5a receptors were blocked by pre-treatment of whole blood with 1 μM PMX205 [28], 37°C, 30 minutes, end-over-end rotation. Bacterial survival was quantified as multiplication factor of number of surviving colonies relative to the starting inoculum. Each strain was cultured in blood from at least three donors and tested in triplicate.

### Neutrophil purification

Neutrophils were purified from fresh human blood using the MACSxpress^®^ Neutrophil Isolation Kit (Miltenyi Biotec) according to manufacturer’s guidelines. Red cells were water-lysed and neutrophils used immediately. All cytometry was carried out on a FACSCalibur cell analyzer (BD Biosciences) and analyzed using FlowJo Software (Treestar, OR).

### Neutrophil phagocytosis assays

For neutrophil uptake studies, GAS were pre-labelled with FITC-conjugated group A carbohydrate antibody (Abcam) and uptake by purified human neutrophils was assayed as described previously [29]. Breifly, GAS were opsonized with fresh human serum (+/- pre-treatment with 0.5 μM rScpA or rAur for 30 minutes at 37°C with end-over-end rotation). Opsonophagocytosis was carried out following incubation of 5x10^5^ neutrophils with opsonized bacteria for 30 minutes at 37°C with end-over-end rotation. The reaction was stopped by addition of stop solution (0.02% EDTA in 0.9% saline) and phagocytosis was quantified as percentage of FITC-positive neutrophils, indicative of bacterial uptake. For surface staining, 5x10^5^ fresh neutrophils were incubated with unlabeled GAS for 30 minutes, 37°C with end-to-end rotation and blocked with Human TruStain FcXTM (Biolegend) for 10 minutes on ice. Surface staining for CD11b was carried out using AF488-conjugated anti-CD11-b (Biolegend 301318) or isotype control antibody (Biolegend 400129) to assess non-specific binding (S1 Table) for 20 minutes on ice, and gMFI ascertained for all samples.

### Neutrophil chemotaxis assay

24-well transwell plates (Corning 3421) were blocked with RPMI (10% FCS) for 30 minutes prior to addition of rScpA-cleaved and uncleaved C3a or C5a (R&D) (+/- pre-treatment with 100 ng rScpA, 37°C, 16 hours). 1x10^6^ purified neutrophils were added to the inner chamber, and incubated for 30 minutes, 37°C, 5% CO_2_. The number of neutrophils which had migrated into the lower chamber were quantified using CountBright™ Absolute Counting Beads (ThermoFisher Scientific).

### Calcium mobilization assay

A flow cytometric method for calcium mobilization was carried out as described previously [53] with minor modifications. Briefly, neutrophils (1x10^7^/ml) were labeled with fluo-4-AM (0.9 μM) in assay buffer (103 mM NaCl, 4.6 mM KCl, 1 mM CaCl_2_, 5 mM glucose, 20 mM HEPES, pH7.4). rScpA-cleaved (100 ng) and uncleaved C3a or C5a (100 ng/ml) (R&D) were added directly to neutrophils, and change in mean fluorescence intensity was measured immediately and recorded over 120 seconds.

### Murine intra-muscular infection

6–8 week old C57BL/6 (Charles River, Margate, UK), and age and weight-matched C57BL/6 C5^-/-^ and C57BL/6 C3^-/-^/C5^-/-^ female mice were challenged intra-muscularly with 1×10^8^ GAS, and quantitative endpoints compared at 3 hours post infection. Mice were euthanized, blood taken by cardiac puncture and infected thigh muscle, spleen, liver, and draining inguinal lymph nodes dissected. All organs were plated to quantify bacterial cfu and systemic dissemination. Additional C57BL/6 mice were challenged intra-muscularly with 1×10^8^ GAS. Whole thighs were formalin-fixed, and paraffin-embedded for histopathology imaging as described previously [24], or homogenized and prepared for western blotting as described above. No animals were excluded from the study and randomization was not necessary as mice were genetically identical and age, weight and sex matched. No blinding was carried out as mice were caged separately to prevent contamination with different GAS strains.

### Cell culture

The human lung epithelial cell line A549 was cultured in RPMI supplemented with 10% FCS, L-glutamine (2 mM), 37°C, 5% CO_2_. Primary human umbilical vein endothelial cells (HUVEC) were obtained from fresh human umbilical cords by collagenase treatment as previously reported [54]. Monolayers were cultured in Medium 199 supplemented with 20% FCS, Heparin (10 U/ml), endothelial cell growth supplement (30 μg/ml, Millipore), L-glutamine (2 mM), Pen/strep (1X) 37°C, 5% CO_2_. Murine cardiac endothelial cell line (MCEC-1) [55] was cultured in DMEM supplemented with 20% FCS, Heparin (10 U/ml), endothelial cell growth supplement (30 μg/ml, Millipore), L-glutamine (2 mM), Pen/strep (1X) at 37°C with 5% CO_2_. Human and murine endothelial cells were cultured in flasks or plates coated with 1% gelatin (Sigma).

### GAS adhesion assays

A549 and HUVEC cells were seeded into 24-well plates and cultured to confluence. GAS were cultured in THB and washed and resuspended in PBS before being inoculated into each well at an MOI of 10. Adhesion assays were carried out following incubation for 30 minutes at 37°C, 5% CO_2_. Cells were washed 3 times with 1x HBSS (Gibco) to remove non-adherent bacteria and cell-associated bacteria were released following lysis of the cell monolayer in sterile water with trypsin-EDTA (Gibco) and quantified by serial dilution and plating onto CBA. To ensure any differences observed in bacterial recovery were not due to variation in cellular internalization, replica experimental wells were set up. Instead of cell lysis, monolayers were treated with gentamicin (100 μg/ml) in RPMI+FCS+L-glut for 2 hours, 37°C, 5% CO_2_. Cells were subsequently washed and lysed as described above and internalized bacteria were quantified as for adhesion assays.

### rScpA binding to HUVEC

HUVEC were cultured to confluence in 96-well plates and fixed in paraformaldehyde (2%), 15 minutes, room temperature. Cells were washed 3 times in PBS and blocked in 1% BSA, 1 hour, room temperature. rScpA was added to cells in PBS or PBS-FCS (10%), in doubling dilutions from 250 ng. Bound protein was detected following incubation with mouse anti-ScpA serum (1:1000) for 2 hours at room temperature and goat-anti mouse-HRP (1:1000) (Abcam) for 1 hour, room temperature. Samples were incubated with 3, 3', 5, 5'-Tetramethylbenzidine (TMB) substrate (Sigma) for 20 minutes, room temperature and reaction stopped following addition of 1 M H_2_S0_4_. Relative levels of rScpA binding were quantified as A_450_ values.

### Statistics

All statistical analyses were performed with GraphPad Prism 5.0. Comparison of two datasets was carried out using unpaired Mann-Whitney U or T-test. A p-value of ≤0.05 was considered significant.

## Supporting information

S1 Fig Expression of recombinant proteins CppA, ScpA and Aur. SDS-PAGE of recombinantly expressed proteins CppA, ScpA, and Aur, demonstrating the purity of preparations. All proteins were eluted or buffer-exchanged into PBS. For CppA and ScpA gels, molecular weight marker lane has been spliced together with relevant recombinant protein lanes as marked.
(TIF)

S2 Fig Specific cleavage of human C3 is not mediated by streptococcal proteins CppA, SPEB, or SpyCEP. Attempted cleavage of human C3 by A) rCppA protein, B) Cell pellets (4x10^6^ cfu) and supernatants from isogenic strains GAS-M49 and GAS-M49_ΔspeB_, and C) Cell pellets (4x10^6^ cfu) of isogenic GAS-M81 and GAS-M81_ΔspyCEP_, following co-incubation for 16 hours, 37°C. For panel B, horizontal line visible in right panel (cell pellets) is lined paper in background.
(TIF)

S3 Fig Generation of GAS M1 and M89 isogenic ScpA knock-out strains. **A) Genetic inactivation of ScpA** was achieved by allelic exchange mutagenesis using suicide vector pUCMUT. Double recombination events between R1 and R2 resulted in replacement of a 1500 bp region of *scpA* containing the putative peptidase domain, including catalytic triad, with kanamycin resistance gene *aphA3* (1500 bp). The cloning strategy was designed such that the size of the resulting *mga* operon transcript would be the same as for the wildtype strain to reduce the risk of polar effects on *mga*. The orientation of *aphA3* and its promoter is opposite to that of the *mga* operon to prevent alteration of transcript levels of adjacent genes. **B-F) Characterization of ScpA allelic exchange mutants**; Comparative growth curves, quantified as optical density at 600 nm, for B) GAS-M1 and GAS-M1_ΔscpA_ and C) GAS-M89, GAS-M89_ΔscpA_, and GAS-M89_ΔscpA_ subsequently complemented with either empty replicative vector, pOri (GAS-M89_ΔpC_) or with replicative vector pOri expressing ScpA (GAS-M89_ΔpCscpA_). Error bars represent mean+/- SD of 3 biological replicates. **D) Visualization of ScpA protein expression by GAS-M89**, **GAS-M89**_**ΔscpA**_, **GAS-M89**_**ΔpC**_ **and GAS-M89**_**ΔpCscpA**_ **by western blot**. ScpA protein in 1 μg bacterial cell wall extract was compared between strains following detection with anti-ScpA mouse serum. **E) Assessment of mutagenesis-induced polar effects on the *mga* operon**. Comparison between GAS-M1 and GAS-M1_ΔscpA_ for effects on expression of *fba* by qPCR (data represent mean +/- SD fold change calculated by ΔΔct method) and ScpA, SIC, and M protein by western blot. **F) Assessment of mutagenesis-induced effects on global regulatory system CovR/S**. To rule out polar effects on the CovR/S regulon, expression of two virulence factors directly regulated by CovR, SpyCEP and the hyaluronic acid capsule, were compared by western blot of cell wall extract and ELISA respectively. (Capsule: mean +/- SD of 4 experimental replicates, ns = p > 0.05).
(TIF)

S4 Fig Physiological and bacterial C3 cleavage sites. Schematic representation of C3 cleavage site by physiological C3 convertase (top panel), and by bacterial C3-ases NalP (*Neisseria meningitidis*) (second panel), Aureolysin (*Staphylococcus aureus*) (third panel), and ScpA (GAS) (bottom panel).
(TIF)

S5 Fig C1q, C2 and C4 were not cleaved by ScpA. GAS-M1 cell pellets (4x10^6^ cfu) or buffer alone (THB) were incubated with each complement factor at 37°C for 16 hours. SDS-PAGE of supernatant following centrifugation was assessed and demonstrated that only complement factor C3 was cleaved by GAS-M1 (cleavage product indicated by black box). Protein chains observed were as expected for all complement factors under denaturing conditions.
(TIF)

S6 Fig Cleavage of C3 by rScpA occurs at a comparable rate to rAur. A) Rate of cleavage of C3 protein by rScpA and rAur over a 4 hour period following incubation of proteins at 1:1 molar ratio in HEPES++ buffer. C3α^scpA^ and C3α^aur^ cleavage products (white arrows) both became detectable after 30 minutes incubation, and became more pronounced after 60 minutes. Duration of incubation in hours is indicated at top of each lane. B) C3 deposition on GAS-M1_ΔscpA_ was compared following incubation with fresh human serum which had been pre-incubated with 0.5 μM of either rScpA or rAur. Data are presented as fluorescence index (FI), calculated as the proportion of positive bacteria expressed as a percentage multiplied by the gMFI. Data represent mean +/- SD of 4 technical replicates, (one-tailed T-test * = p < 0.05).
(TIF)

S7 Fig C3 protein is not degraded in human serum following heat-inactivation. Fresh human serum was heat-inactivated (HI) by incubation at 56°C for 30 minutes. Degradation of C3 in 1% heat-inactivated serum was compared with 1% fresh serum from the same donor, and C3-depleted serum, and assessed by western blot analysis. Expected C3 α and β chains were observed for fresh and heat-inactivated serum under denaturing conditions, demonstrating C3 was intact following heat-inactivation.
(TIF)

S8 Fig ScpA cleaves murine complement factors. A-B) Cleavage of murine complement factors A) mC3a and B) mC5a by whole cell pellets of GAS-M1 vs GAS-M1_ΔscpA_ and rScpA (100 ng). Only GAS-M1 expressing ScpA was able to cleave these complement factors. Cleaved moieties (C3a^scpA^, C5a^scpA^) are indicated by a white arrow in each panel. C-D) Rate of rScpA cleavage of complement components was assessed over 60 minutes at 37°C and visualized by SDS-PAGE and Coomassie staining. C) Cleavage of mC3a by rScpA at 10:1 molar ratio. D) Cleavage of mC5a by rScpA at 10:1 molar ratio. Duration of incubation in minutes is indicated above each lane.
(TIF)

S9 Fig ScpA is expressed during murine intra-muscular infection. Expression analysis of ScpA *in vivo*. C57BL/6 mice were infected intra-muscularly with GAS-M1 or GAS-M1_ΔscpA_ (n = 4/group). ScpA expression in 10 μg thigh tissue homogenate was compared between individual mice following immunoblot with anti-ScpA mouse serum. The band unique to GAS-M1 infected mice at 110 kDa is ScpA.
(TIF)

S10 Fig Streptococcal dissemination *in vivo* at 24 hours. Characterization of GAS-M1 and GAS-M1_ΔscpA_ dissemination from the site of infection in a murine model of soft tissue infection in wildtype C57BL/6 mice (n = 8/group) at 24 hour timepoint. Mice were infected intra-muscularly in the thigh. A) Bacteria at the site of infection, B) local spread to the draining inguinal lymph node, and subsequent systemic dissemination to C) spleen, D) liver and E) blood were assessed by quantitative culture. Each data point indicates one mouse and line depicts median value. (Mann Whitney U, * = p<0.05).
(TIF)

S11 Fig Early pathological changes at the site of infection in a murine model. Haemotoxylin and eosin stained tissue sections of whole thigh obtained from C57BL/6 mice 3 hours after intra-muscular infection with GAS-M1 or GAS-M1_ΔscpA_ (n = 4/group). A) Magnification x10. White arrows indicate regions with detectable GAS. Contiguous sections were Gram stained to aid detection of bacteria. B) Higher power (40x) images of sections with detectable bacteria from mice infected with GAS-M1 (n = 3/4) and GAS-M1_ΔscpA_ (n = 1/4). White arrows indicate regions with detectable GAS. Note very limited but necrotic leukocyte infiltrate.
(TIF)

S12 Fig ScpA does not mediate internalization of GAS by epithelial or endothelial cells. A+B) Internalization of A) GAS-M1 and B) GAS-M89 and isogenic ΔscpA strains by A549 epithelial cells was compared by quantitative culture (30 min incubation). Data represent mean+/-SD of 3 experimental replicates. C+D) Internalization of C) GAS-M1 and D) GAS-M89 and isogenic ΔscpA strains by primary human HUVEC was compared by quantitative culture (30 min incubation). Data represent mean+/-SD of 3 experimental replicates.
(TIF)

S13 Fig ScpA is a member of the CovR and RocA regulon. Western blot comparing expression of ScpA between pairs of isogenic strains expressing active or inactive CovR or RocA (strains listed in Table 1). ScpA expression in 1 μg bacterial cell wall extract was compared between isogenic strains following detection with anti-ScpA mouse serum. Functional CovR and RocA repressed expression of ScpA.
(TIF)

S1 Table Flow cytometry CD11b antibody specificity (4 technical replicates, representative of 3 donors). (PDF)

## References

[1] Agarwal V, Hammerschmidt S, Malm S, Bergmann S, Riesbeck K, Blom AM (2012) Enolase of Streptococcus pneumoniae binds human complement inhibitor C4b-binding protein and contributes to complement evasion. J Immunol 189: 3575–3584. doi: 10.4049/jimmunol.1102934 22925928 10.4049/jimmunol.1102934

[2] Ermert D, Weckel A, Agarwal V, Frick IM, Bjorck L, Blom AM (2013) Binding of complement inhibitor C4b-binding protein to a highly virulent Streptococcus pyogenes M1 strain is mediated by protein H and enhances adhesion to and invasion of endothelial cells. J Biol Chem 288: 32172–32183. doi: 10.1074/jbc.M113.502955 24064215 10.1074/jbc.M113.502955 PMC3820857

[3] Hair PS, Echague CG, Sholl AM, Watkins JA, Geoghegan JA, Foster TJ et al (2010) Clumping factor A interaction with complement factor I increases C3b cleavage on the bacterial surface of Staphylococcus aureus and decreases complement-mediated phagocytosis. Infect Immun 78: 1717–1727. doi: 10.1128/IAI.01065-09 20100856 10.1128/IAI.01065-09 PMC2849425

[4] Honda-Ogawa M, Ogawa T, Terao Y, Sumitomo T, Nakata M, Ikebe K et al (2013) Cysteine proteinase from Streptococcus pyogenes enables evasion of innate immunity via degradation of complement factors. J Biol Chem 288: 15854–15864. doi: 10.1074/jbc.M113.469106 23589297 10.1074/jbc.M113.469106 PMC3668742

[5] Jongerius I, Kohl J, Pandey MK, Ruyken M, van Kessel KP, van Strijp JA et al (2007) Staphylococcal complement evasion by various convertase-blocking molecules. J Exp Med 204: 2461–2471. doi: 10.1084/jem.20070818 17893203 10.1084/jem.20070818 PMC2118443

[6] Jusko M, Potempa J, Kantyka T, Bielecka E, Miller HK, Kalinska M et al (2014) Staphylococcal proteases aid in evasion of the human complement system. J Innate Immun 6: 31–46. doi: 10.1159/000351458 23838186 10.1159/000351458 PMC3972074

[7] Pandiripally V, Gregory E, Cue D (2002) Acquisition of regulators of complement activation by Streptococcus pyogenes serotype M1. Infect Immun 70: 6206–6214. doi: 10.1128/IAI.70.11.6206-6214.2002 12379699 10.1128/IAI.70.11.6206-6214.2002 PMC130388

[8] Carapetis JR, Steer AC, Mulholland EK, Weber M (2005) The global burden of group A streptococcal diseases. Lancet Infect Dis 5: 685–694. doi: 10.1016/S1473-3099(05)70267-X 16253886 10.1016/S1473-3099(05)70267-X

[9] Carlsson F, Sandin C, Lindahl G (2005) Human fibrinogen bound to Streptococcus pyogenes M protein inhibits complement deposition via the classical pathway. Mol Microbiol 56: 28–39. doi: 10.1111/j.1365-2958.2005.04527.x 15773976 10.1111/j.1365-2958.2005.04527.x

[10] Cleary PP, Prahbu U, Dale JB, Wexler DE, Handley J (1992) Streptococcal C5a peptidase is a highly specific endopeptidase. Infect Immun 60: 5219–5223. 1452354 10.1128/iai.60.12.5219-5223.1992 PMC258300

[11] Terao Y, Mori Y, Yamaguchi M, Shimizu Y, Ooe K, Hamada S et al (2008) Group A streptococcal cysteine protease degrades C3 (C3b) and contributes to evasion of innate immunity. J Biol Chem 283: 6253–6260. doi: 10.1074/jbc.M704821200 18160402 10.1074/jbc.M704821200

[12] Laarman AJ, Ruyken M, Malone CL, van Strijp JA, Horswill AR, Rooijakkers SH (2011) Staphylococcus aureus metalloprotease aureolysin cleaves complement C3 to mediate immune evasion. J Immunol 186: 6445–6453. doi: 10.4049/jimmunol.1002948 21502375 10.4049/jimmunol.1002948

[13] Janssen BJC, Huizinga EG, Raaijmakers HCA, Roos A, Daha MR, Nilsson-Ekdahl K et al (2005) Structures of complement component C3 provide insights into the function and evolution of immunity. Nature 437: 500–11 doi: 10.1038/nature04005 16177781 10.1038/nature04005

[14] Turner CE, Dryden M, Holden MT, Davies FJ, Lawrenson RA, Farzaneh L, et al (2013) Molecular analysis of an outbreak of lethal postpartum sepsis caused by Streptococcus pyogenes. J Clin Microbiol 51: 2089–2095. doi: 10.1128/JCM.00679-13 23616448 10.1128/JCM.00679-13 PMC3697669

[15] O'Connor SP, Cleary PP (1986) Localization of the streptococcal C5a peptidase to the surface of group A streptococci. Infect Immun 53: 432–434. 3525415 10.1128/iai.53.2.432-434.1986 PMC260894

[16] Stafslien DK, Cleary PP (2000) Characterization of the streptococcal C5a peptidase using a C5a-green fluorescent protein fusion protein substrate. J Bacteriol 182: 3254–3258. 10809707 10.1128/jb.182.11.3254-3258.2000 PMC94514

[17] Ji Y, Carlson B, Kondagunta A, Cleary PP (1997) Intranasal immunization with C5a peptidase prevents nasopharyngeal colonization of mice by the group A Streptococcus. Infect Immun 65: 2080–2087. 9169735 10.1128/iai.65.6.2080-2087.1997 PMC175287

[18] Ji Y, Schnitzler N, DeMaster E, Cleary P (1998) Impact of M49, Mrp, Enn, and C5a peptidase proteins on colonization of the mouse oral mucosa by Streptococcus pyogenes. Infect Immun 66: 5399–5405. 9784550 10.1128/iai.66.11.5399-5405.1998 PMC108676

[19] Ji Y, McLandsborough L, Kondagunta A, Cleary PP (1996) C5a peptidase alters clearance and trafficking of group A streptococci by infected mice. Infect Immun 64: 503–510. 8550199 10.1128/iai.64.2.503-510.1996 PMC173793

[20] Kagawa TF, O'Connell MR, Mouat P, Paoli M, O'Toole PW, Cooney JC (2009) Model for substrate interactions in C5a peptidase from Streptococcus pyogenes: A 1.9 A crystal structure of the active form of ScpA. J Mol Biol 386: 754–772. doi: 10.1016/j.jmb.2008.12.074 19152799 10.1016/j.jmb.2008.12.074

[21] Lynskey NN, Goulding D, Gierula M, Turner CE, Dougan G, Edwards RJ et al (2013) RocA truncation underpins hyper-encapsulation, carriage longevity and transmissibility of serotype M18 group A streptococci. PLoS Pathog 9: e1003842 doi: 10.1371/journal.ppat.1003842 24367267 10.1371/journal.ppat.1003842 PMC3868526

[22] Angel CS, Ruzek M, Hostetter MK (1994) Degradation of C3 by Streptococcus pneumoniae. J Infect Dis 170: 600–608. 8077717 10.1093/infdis/170.3.600

[23] Edwards RJ, Taylor GW, Ferguson M, Murray S, Rendell N, Wrigley A, et al (2005) Specific C-terminal cleavage and inactivation of interleukin-8 by invasive disease isolates of Streptococcus pyogenes. J Infect Dis 192(5):783–90 doi: 10.1086/432485 16088827 10.1086/432485

[24] Kurupati P, Turner CE, Tziona I, Lawrenson RA, Alam FM, Nohadani M et al (2010) Chemokine-cleaving Streptococcus pyogenes protease SpyCEP is necessary and sufficient for bacterial dissemination within soft tissues and the respiratory tract. Mol Microbiol 76: 1387–1397. doi: 10.1111/j.1365-2958.2010.07065.x 20158613 10.1111/j.1365-2958.2010.07065.x PMC2904501

[25] Bajic G, Yatime L, Klos A, Andersen GR (2013) Human C3a and C3a desArg anaphylatoxins have conserved structures, in contrast to C5a and C5a desArg. Protein Sci 22: 204–212. doi: 10.1002/pro.2200 23184394 10.1002/pro.2200 PMC3588916

[26] Del TE, Vacca I, Ram S, Rappuoli R, Serruto D (2014) Neisseria meningitidis NalP cleaves human complement C3, facilitating degradation of C3b and survival in human serum. Proc Natl Acad Sci U S A 111: 427–432. doi: 10.1073/pnas.1321556111 24367091 10.1073/pnas.1321556111 PMC3890809

[27] Sanderson-Smith M, De Oliveira DM, Guglielmini J, McMillan DJ, Vu T, Holien JK et al (2014) A systematic and functional classification of Streptococcus pyogenes that serves as a new tool for molecular typing and vaccine development. J Infect Dis 210(8):1325–38 doi: 10.1093/infdis/jiu260 24799598 10.1093/infdis/jiu260 PMC6083926

[28] March DR, Proctor LM, Stoermer MJ, Sbaglia R, Abbenante G, Reid RC et al (2004) Potent cyclic antagonists of the complement C5a receptor on human polymorphonuclear leukocytes. Relationships between structures and activity. Mol Pharmacol 65(4):868–79 doi: 10.1124/mol.65.4.868 15044616 10.1124/mol.65.4.868

[29] Lynskey NN, Banerji S, Johnson LA, Holder KA, Reglinski M, Wing PA et al (2015) Rapid Lymphatic Dissemination of Encapsulated Group A Streptococci via Lymphatic Vessel Endothelial Receptor-1 Interaction. PLoS Pathog 11: e1005137 doi: 10.1371/journal.ppat.1005137 26352587 10.1371/journal.ppat.1005137 PMC4564194

[30] Cheng Q, Stafslien D, Purushothaman SS, Cleary P (2002) The group B streptococcal C5a peptidase is both a specific protease and an invasin. Infect Immun 70(5):2408–13 doi: 10.1128/IAI.70.5.2408-2413.2002 11953377 10.1128/IAI.70.5.2408-2413.2002 PMC127948

[31] Beckmann C, Waggoner JD, Harris TO, Tamura GS, Rubens CE (2002) Identification of novel adhesins from Group B streptococci by use of phage display reveals that C5a peptidase mediates fibronectin binding. Infect Immun 70(6):2869–76 doi: 10.1128/IAI.70.6.2869-2876.2002 12010974 10.1128/IAI.70.6.2869-2876.2002 PMC128012

[32] Purushothaman SS, Park HS, Cleary PP (2004) Promotion of fibronectin independent invasion by C5a peptidase into epithelial cells in group A Streptococcus. Indian J Med Res 119 Suppl:44–7 15232161

[33] Jarva H, Janulczyk R, Hellwage J, Zipfel PF, Bjorck L, Meri S (2002) Streptococcus pneumoniae evades complement attack and opsonophagocytosis by expressing the pspC locus-encoded Hic protein that binds to short consensus repeats 8–11 of factor H. J Immunol 168: 1886–1894. 11823523 10.4049/jimmunol.168.4.1886

[34] Rooijakkers SH, Ruyken M, Roos A, Daha MR, Presanis JS, Sim RB et al (2005) Immune evasion by a staphylococcal complement inhibitor that acts on C3 convertases. Nat Immunol 6: 920–927. doi: 10.1038/ni1235 16086019 10.1038/ni1235

[35] Brown CK, Gu ZY, Matsuka YV, Purushothaman SS, Winter LA, Cleary PP et al (2005) Structure of the streptococcal cell wall C5a peptidase. Proc Natl Acad Sci U S A 102: 18391–18396. doi: 10.1073/pnas.0504954102 16344483 10.1073/pnas.0504954102 PMC1317908

[36] Cheng Q, Stafslien D, Purushothaman SS, Cleary P (2002) The group B streptococcal C5a peptidase is both a specific protease and an invasin. Infect Immun 70: 2408–2413. doi: 10.1128/IAI.70.5.2408-2413.2002 11953377 10.1128/IAI.70.5.2408-2413.2002 PMC127948

[37] Chmouryguina I, Suvorov A, Ferrieri P, Cleary PP (1996) Conservation of the C5a peptidase genes in group A and B streptococci. Infect Immun 64: 2387–2390. 8698456 10.1128/iai.64.7.2387-2390.1996 PMC174087

[38] Cleary PP, Peterson J, Chen C, Nelson C (1991) Virulent human strains of group G streptococci express a C5a peptidase enzyme similar to that produced by group A streptococci. Infect Immun 59: 2305–2310. 2050400 10.1128/iai.59.7.2305-2310.1991 PMC258011

[39] Cheng Q, Carlson B, Pillai S, Eby R, Edwards L, Olmsted SB et al (2001) Antibody against surface-bound C5a peptidase is opsonic and initiates macrophage killing of group B streptococci. Infect Immun 69: 2302–2308. doi: 10.1128/IAI.69.4.2302-2308.2001 11254587 10.1128/IAI.69.4.2302-2308.2001 PMC98159

[40] Cleary PP, Matsuka YV, Huynh T, Lam H, Olmsted SB (2004) Immunization with C5a peptidase from either group A or B streptococci enhances clearance of group A streptococci from intranasally infected mice. Vaccine 22: 4332–4341. doi: 10.1016/j.vaccine.2004.04.030 15474726 10.1016/j.vaccine.2004.04.030

[41] Martins TB, Hoffman JL, Augustine NH, Phansalkar AR, Fischetti VA, Zabriskie JB, et al (2008) Comprehensive analysis of antibody responses to streptococcal and tissue antigens in patients with acute rheumatic fever. Int Immunol 20: 445–452. doi: 10.1093/intimm/dxn004 18245783 10.1093/intimm/dxn004

[42] O'Connor SP, Darip D, Fraley K, Nelson CM, Kaplan EL, Cleary PP (1991) The human antibody response to streptococcal C5a peptidase. J Infect Dis 163: 109–116. 1984457 10.1093/infdis/163.1.109

[43] Shet A, Kaplan EL, Johnson DR, Cleary PP (2003) Immune response to group A streptococcal C5a peptidase in children: implications for vaccine development. J Infect Dis 188: 809–817. doi: 10.1086/377700 12964111 10.1086/377700

[44] Park SY, Shin YP, Kim CH, Park HJ, Seong YS, Kim BS et al (2008) Immune evasion of Enterococcus faecalis by an extracellular gelatinase that cleaves C3 and iC3b. J Immunol 181: 6328–6336. 18941224 10.4049/jimmunol.181.9.6328

[45] Sriskandan S, Unnikrishnan M, Krausz T, Cohen J (2000) Mitogenic factor (MF) is the major DNase of serotype M89 Streptococcus pyogenes. Microbiology 146 (Pt 11): 2785–2792. doi: 10.1099/00221287-146-11-2785 11065357 10.1099/00221287-146-11-2785

[46] Lukomski S, Sreevatsan S, Amberg C, Reichardt W, Woischnik M, Podbielski A et al (1997) Inactivation of Streptococcus pyogenes extracellular cysteine protease significantly decreases mouse lethality of serotype M3 and M49 strains. J Clin Invest. 1997 6 1;99(11):2574–80. doi: 10.1172/JCI119445 9169486 10.1172/JCI119445 PMC508102

[47] Holden MT, Heather Z, Paillot R, Steward KF, Webb K, Ainslie F et al (2009) Genomic evidence for the evolution of Streptococcus equi: host restriction, increased virulence, and genetic exchange with human pathogens. PLoS Pathog 3;5(3):e1000346 doi: 10.1371/journal.ppat.1000346 19325880 10.1371/journal.ppat.1000346 PMC2654543

[48] Turner C. E., Kurupati P., Jones M. D., Edwards R. J., & Sriskandan S. (2009) Emerging role of the IL-8 cleaving enzyme SpyCEP in Clinical *Streptococcus pyogenes* infection. The Journal of Infectious Diseases, 200(4), 555–563. http://doi.org/10.1086/603541 19591574 10.1086/603541 PMC2820315

[49] Reglinski M, Lynskey NN, Choi YJ, Edwards RJ, Sriskandan S (2016) Development of a multicomponent vaccine for Streptococcus pyogenes based on the antigenic targets of IVIG. J Infect 72: 450–459. doi: 10.1016/j.jinf.2016.02.002 26880087 10.1016/j.jinf.2016.02.002 PMC4796040

[50] Reglinski M, Gierula M, Lynskey NN, Edwards RJ, Sriskandan S (2015) Identification of the Streptococcus pyogenes surface antigens recognised by pooled human immunoglobulin. Sci Rep 5: 15825 doi: 10.1038/srep15825 26508447 10.1038/srep15825 PMC4623672

[51] Brown JS, Hussell T, Gilliland SM, Holden DW, Paton JC, Ehrenstein MR et al (2002) The classical pathway is the dominant complement pathway required for innate immunity to Streptococcus pneumoniae infection in mice. Proc Natl Acad Sci U S A 99: 16969–16974. doi: 10.1073/pnas.012669199 12477926 10.1073/pnas.012669199 PMC139253

[52] Hyams C, Trzcinski K, Camberlein E, Weinberger DM, Chimalapati S, Noursadeghi M et al (2013) Streptococcus pneumoniae capsular serotype invasiveness correlates with the degree of factor H binding and opsonization with C3b/iC3b. Infect Immun 81(1):354–63 doi: 10.1128/IAI.00862-12 23147038 10.1128/IAI.00862-12 PMC3536142

[53] Bernhagen J, Krohn R, Lue H, Gregory JL, Zernecke A, Koenen RR et al (2007) MIF is a noncognate ligand of CXC chemokine receptors in inflammatory and atherogenic cell recruitment. Nat Med 13: 587–596. doi: 10.1038/nm1567 17435771 10.1038/nm1567

[54] Jaffe EA, Nachman RL, Becker CG, Minick CR (1973) Culture of human endothelial cells derived from umbilical veins. Identification by morphologic and immunologic criteria. J Clin Invest 52(11):2745–56 doi: 10.1172/JCI107470 4355998 10.1172/JCI107470 PMC302542

[55] Lidington EA, Rao RM, Marelli-Berg FM, Jat PS, Haskard DO, Mason JC (2002) Conditional immortalization of growth factor-responsive cardiac endothelial cells from H-2Kb-tsA58 mice. Am J Physiol Cell Physiol 282: C67–C74 11742799 10.1152/ajpcell.2002.282.1.C67

