# Supplementary figures and images for: Multi-functional mechanisms of immune evasion by the streptococcal complement inhibitor C5a peptidase
Source: PLoS Pathog. 2017 Aug 14;13(8):e1006493. doi: 10.1371/journal.ppat.1006493 (PMC5555575; doi:10.1371/journal.ppat.1006493)

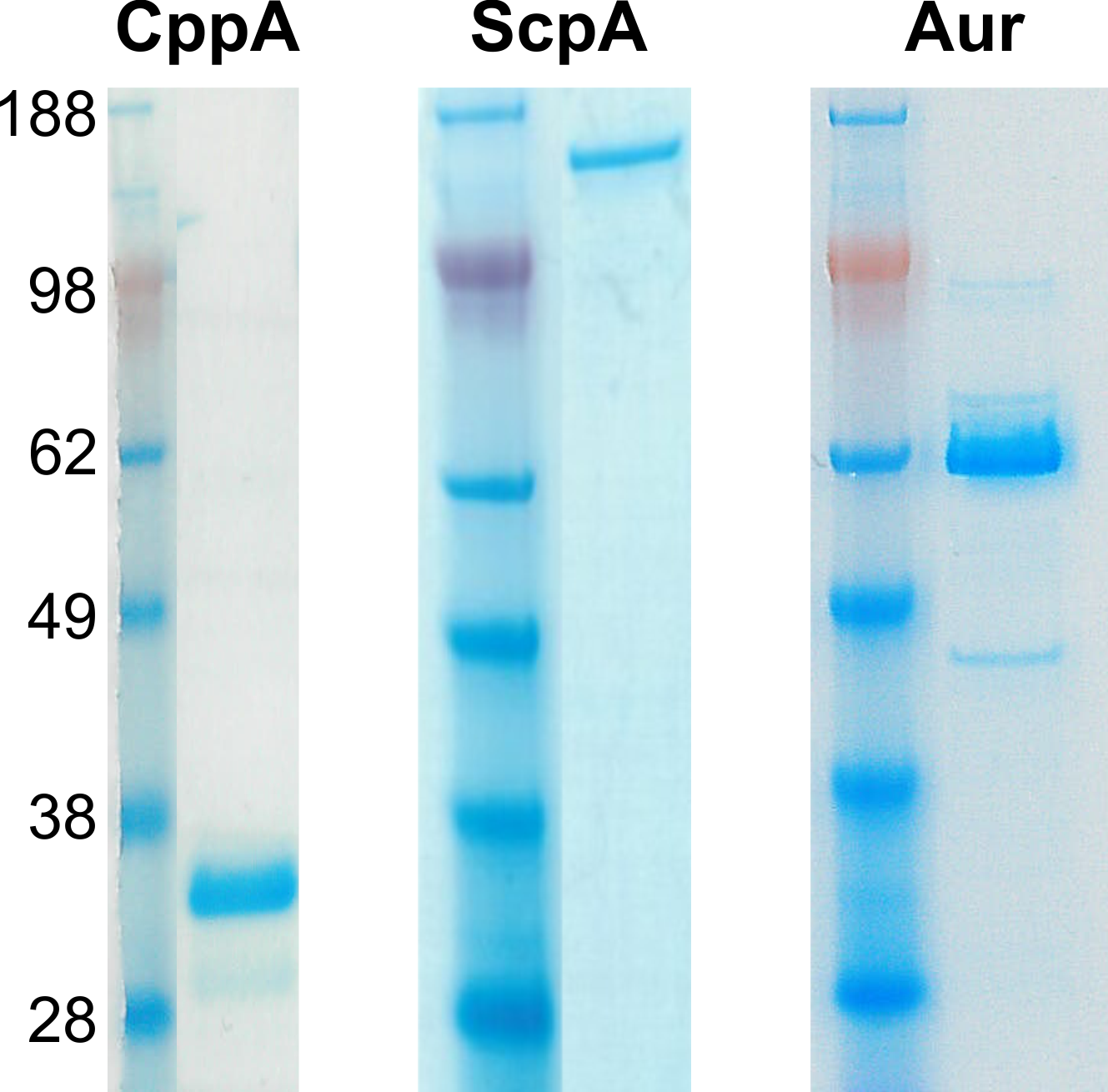

Supplement: S1 Fig — SDS-PAGE of recombinantly expressed proteins CppA, ScpA, and Aur, demonstrating the purity of preparations. All proteins were eluted or buffer-exchanged into PBS. For CppA and ScpA gels, molecular weight marker lane has been spliced together with relevant recombinant protein lanes as marked. (TIF) [file ppat.1006493.s001.tif]

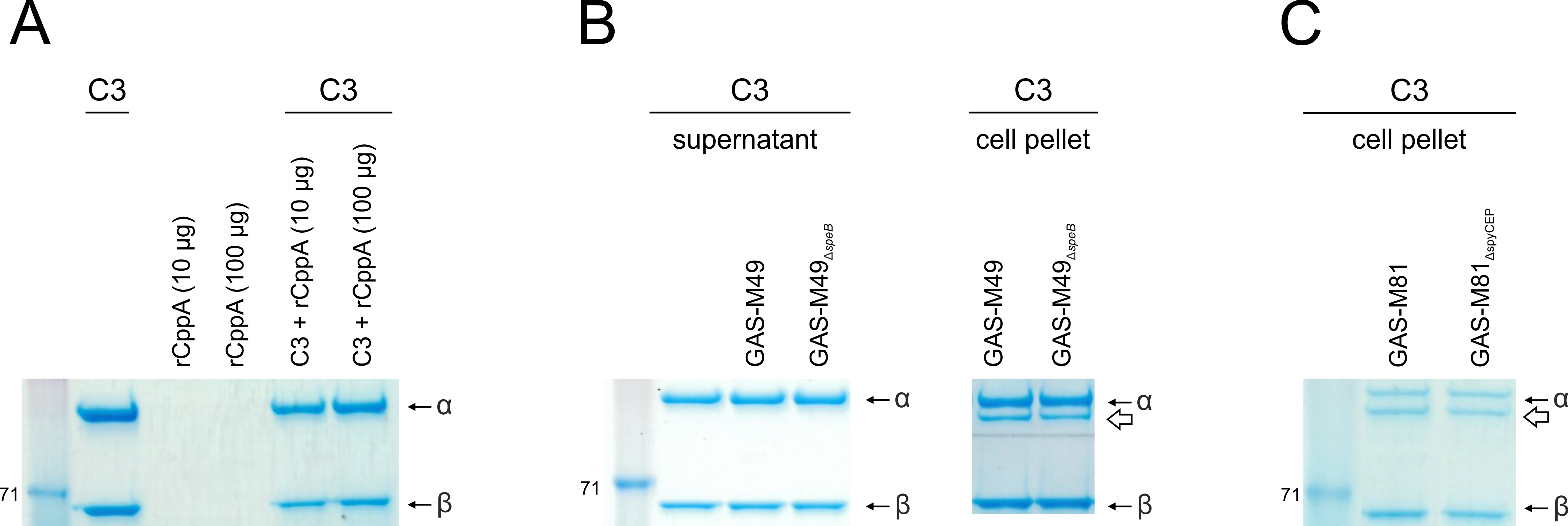

Supplement: S2 Fig — Attempted cleavage of human C3 by A) rCppA protein, B) Cell pellets (4x10^6^ cfu) and supernatants from isogenic strains GAS-M49 and GAS-M49ΔspeB, and C) Cell pellets (4x10^6^ cfu) of isogenic GAS-M81 and GAS-M81ΔspyCEP, following co-incubation for 16 hours, 37°C. For panel B, horizontal line visible in right panel (cell pellets) is lined paper in background. (TIF) [file ppat.1006493.s002.tif]

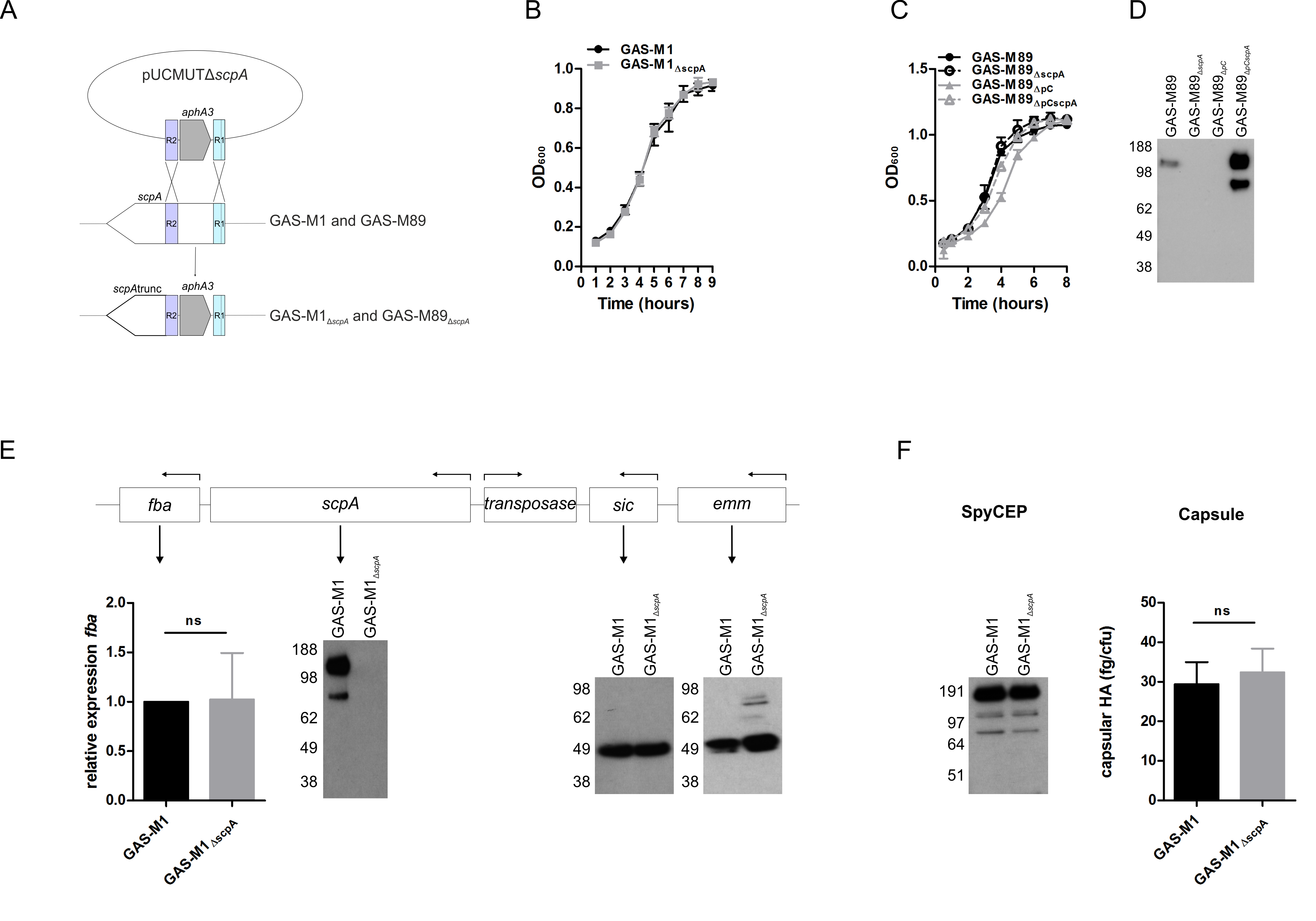

Supplement: S3 Fig — A) Genetic inactivation of ScpA was achieved by allelic exchange mutagenesis using suicide vector pUCMUT. Double recombination events between R1 and R2 resulted in replacement of a 1500 bp region of scpA containing the putative peptidase domain, including catalytic triad, with kanamycin resistance gene aphA3 (1500 bp). The cloning strategy was designed such that the size of the resulting mga operon transcript would be the same as for the wildtype strain to reduce the risk of polar effects on mga. The orientation of aphA3 and its promoter is opposite to that of the mga operon to prevent alteration of transcript levels of adjacent genes. B-F) Characterization of ScpA allelic exchange mutants; Comparative growth curves, quantified as optical density at 600 nm, for B) GAS-M1 and GAS-M1ΔscpA and C) GAS-M89, GAS-M89ΔscpA, and GAS-M89ΔscpA subsequently complemented with either empty replicative vector, pOri (GAS-M89ΔpC) or with replicative vector pOri expressing ScpA (GAS-M89ΔpCscpA). Error bars represent mean+/- SD of 3 biological replicates. D) Visualization of ScpA protein expression by GAS-M89, GAS-M89ΔscpA, GAS-M89ΔpC and GAS-M89ΔpCscpA by western blot. ScpA protein in 1 μg bacterial cell wall extract was compared between strains following detection with anti-ScpA mouse serum. E) Assessment of mutagenesis-induced polar effects on the mga operon. Comparison between GAS-M1 and GAS-M1ΔscpA for effects on expression of fba by qPCR (data represent mean +/- SD fold change calculated by ΔΔct method) and ScpA, SIC, and M protein by western blot. F) Assessment of mutagenesis-induced effects on global regulatory system CovR/S. To rule out polar effects on the CovR/S regulon, expression of two virulence factors directly regulated by CovR, SpyCEP and the hyaluronic acid capsule, were compared by western blot of cell wall extract and ELISA respectively. (Capsule: mean +/- SD of 4 experimental replicates, ns = p > 0.05). (TIF) [file ppat.1006493.s003.tif]

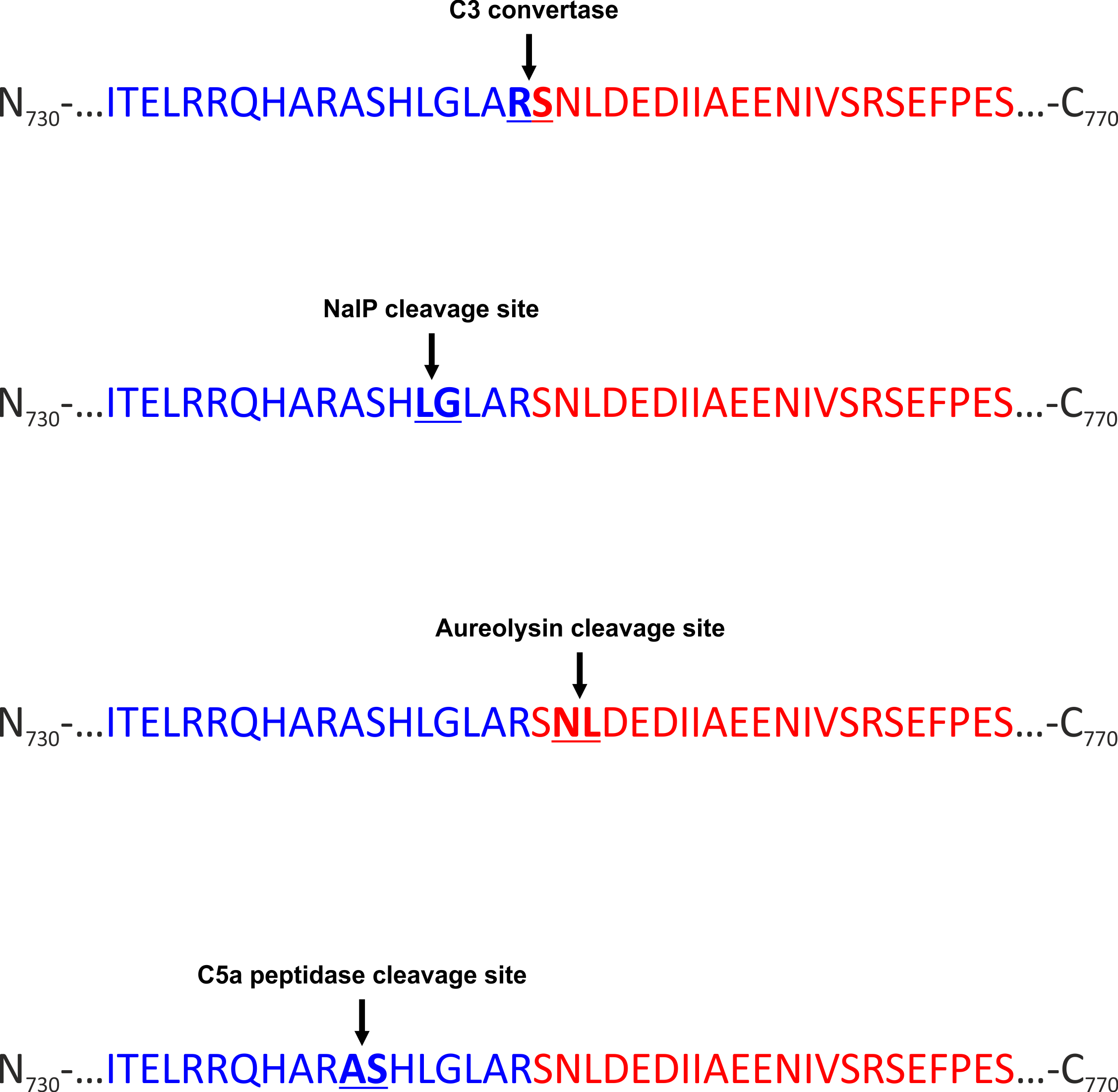

Supplement: S4 Fig — Schematic representation of C3 cleavage site by physiological C3 convertase (top panel), and by bacterial C3-ases NalP (Neisseria meningitidis) (second panel), Aureolysin (Staphylococcus aureus) (third panel), and ScpA (GAS) (bottom panel). (TIF) [file ppat.1006493.s004.tif]

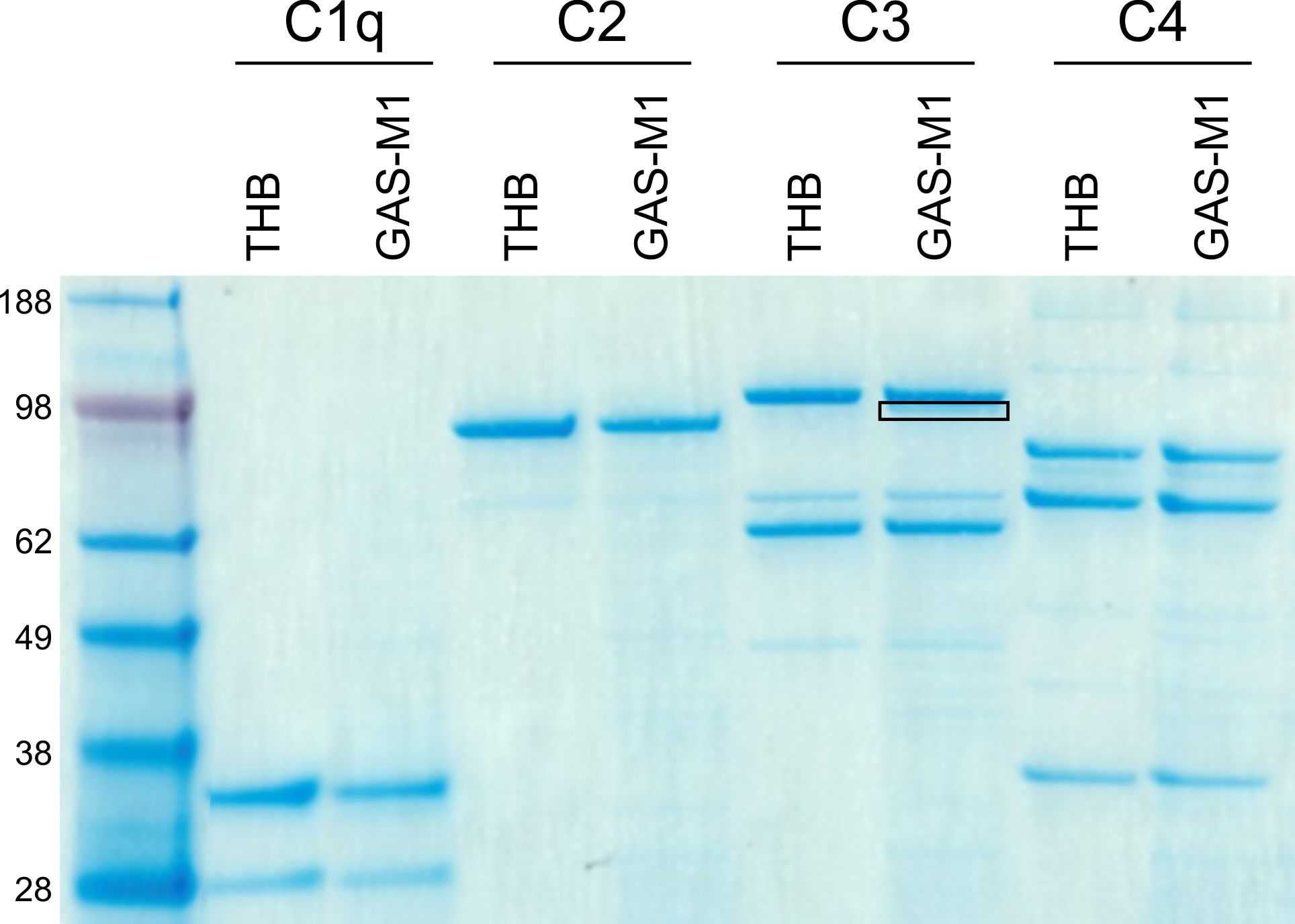

Supplement: S5 Fig — GAS-M1 cell pellets (4x10^6^ cfu) or buffer alone (THB) were incubated with each complement factor at 37°C for 16 hours. SDS-PAGE of supernatant following centrifugation was assessed and demonstrated that only complement factor C3 was cleaved by GAS-M1 (cleavage product indicated by black box). Protein chains observed were as expected for all complement factors under denaturing conditions. (TIF) [file ppat.1006493.s005.tif]

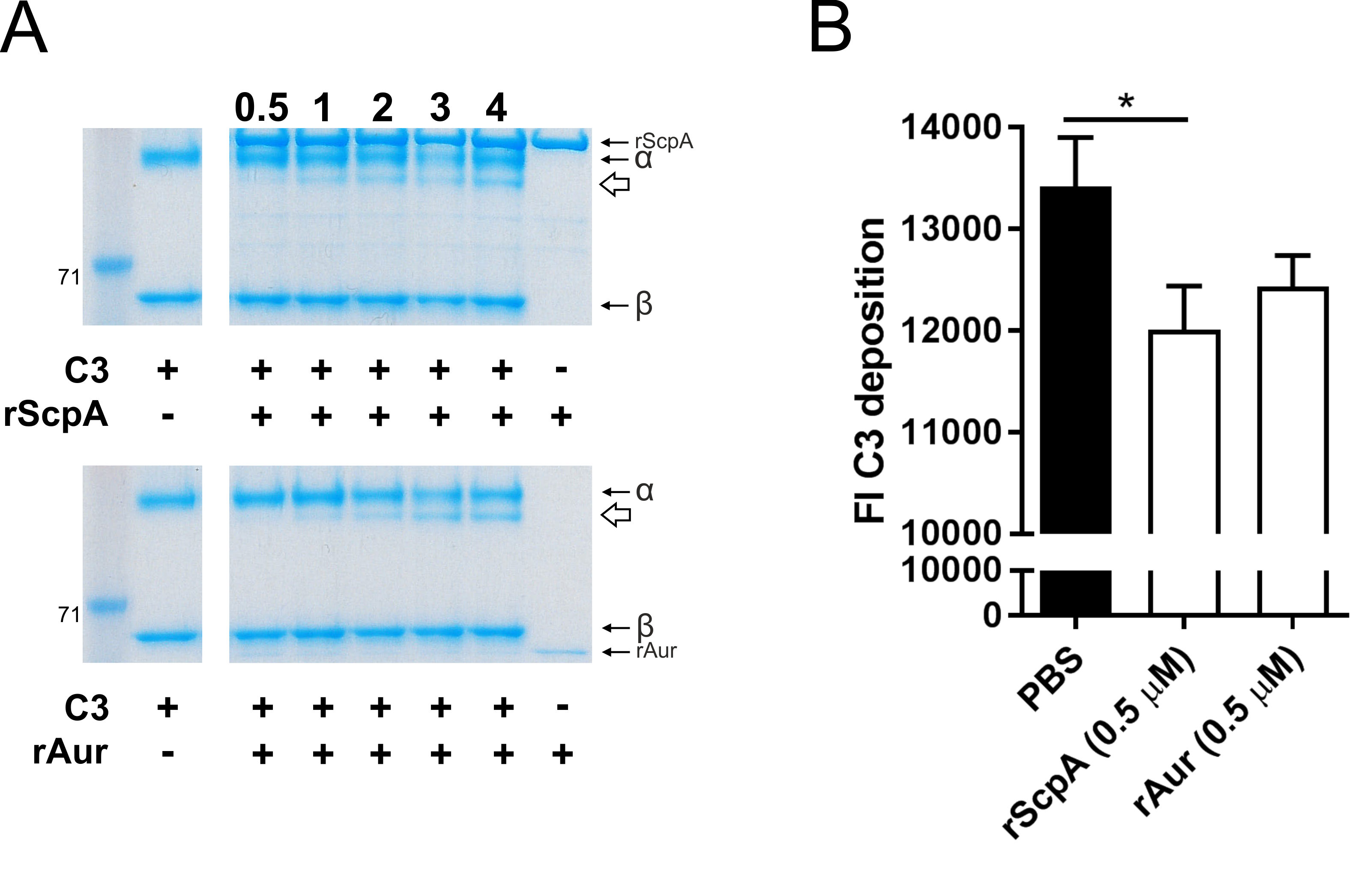

Supplement: S6 Fig — A) Rate of cleavage of C3 protein by rScpA and rAur over a 4 hour period following incubation of proteins at 1:1 molar ratio in HEPES++ buffer. C3αscpA and C3αaur cleavage products (white arrows) both became detectable after 30 minutes incubation, and became more pronounced after 60 minutes. Duration of incubation in hours is indicated at top of each lane. B) C3 deposition on GAS-M1ΔscpA was compared following incubation with fresh human serum which had been pre-incubated with 0.5 μM of either rScpA or rAur. Data are presented as fluorescence index (FI), calculated as the proportion of positive bacteria expressed as a percentage multiplied by the gMFI. Data represent mean +/- SD of 4 technical replicates, (one-tailed T-test * = p < 0.05). (TIF) [file ppat.1006493.s006.tif]

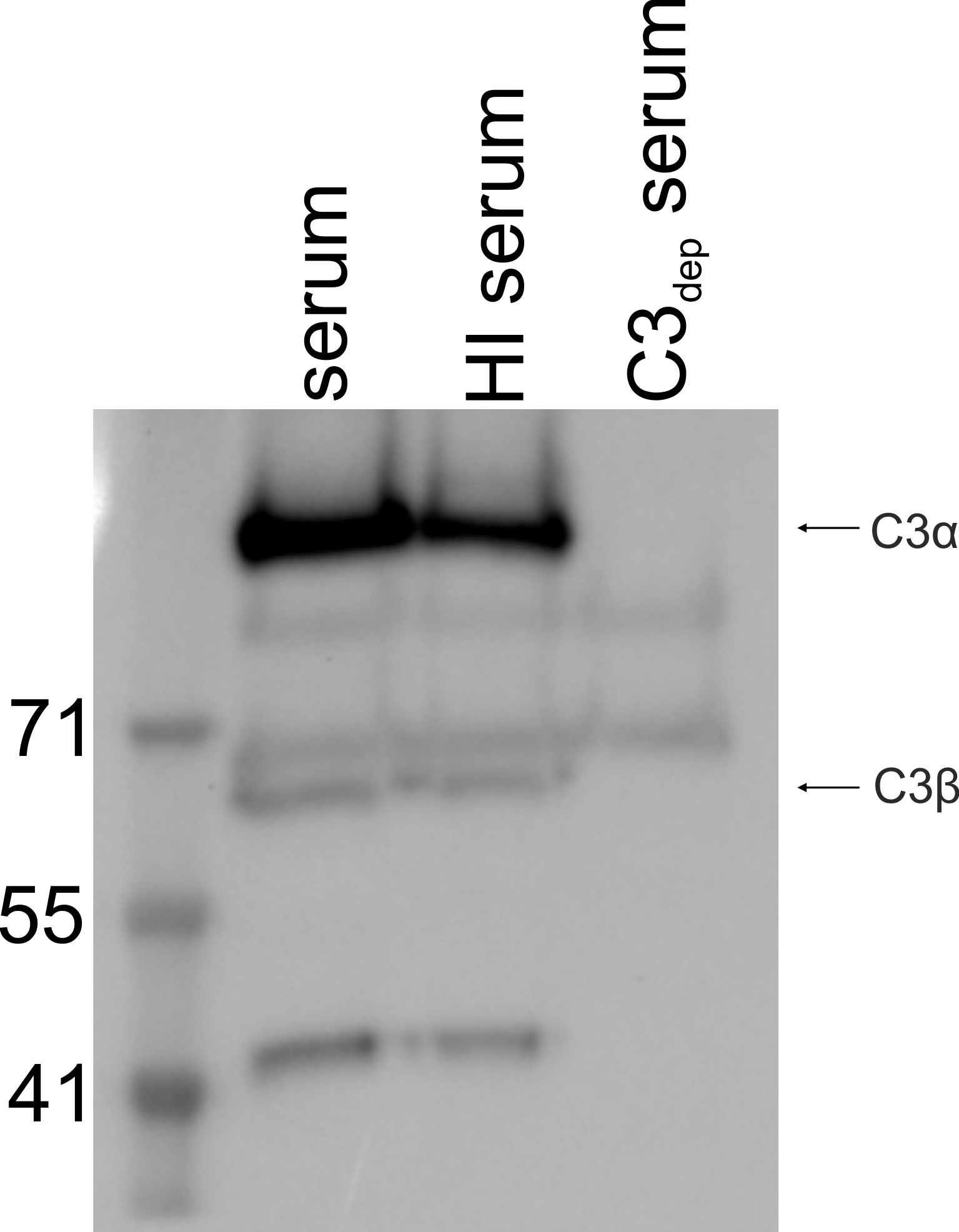

Supplement: S7 Fig — Fresh human serum was heat-inactivated (HI) by incubation at 56°C for 30 minutes. Degradation of C3 in 1% heat-inactivated serum was compared with 1% fresh serum from the same donor, and C3-depleted serum, and assessed by western blot analysis. Expected C3 α and β chains were observed for fresh and heat-inactivated serum under denaturing conditions, demonstrating C3 was intact following heat-inactivation. (TIF) [file ppat.1006493.s007.tif]

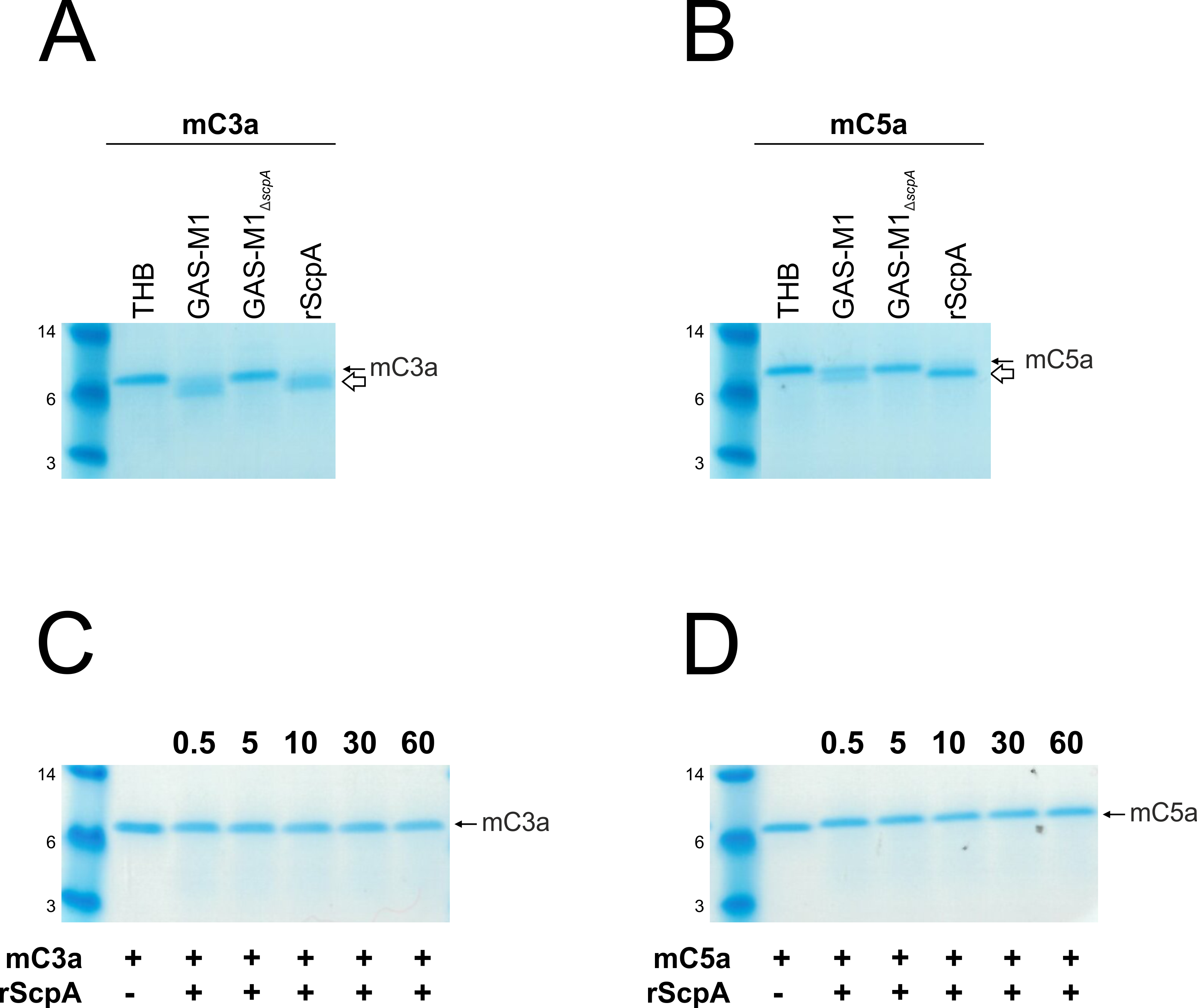

Supplement: S8 Fig — A-B) Cleavage of murine complement factors A) mC3a and B) mC5a by whole cell pellets of GAS-M1 vs GAS-M1ΔscpA and rScpA (100 ng). Only GAS-M1 expressing ScpA was able to cleave these complement factors. Cleaved moieties (C3ascpA, C5ascpA) are indicated by a white arrow in each panel. C-D) Rate of rScpA cleavage of complement components was assessed over 60 minutes at 37°C and visualized by SDS-PAGE and Coomassie staining. C) Cleavage of mC3a by rScpA at 10:1 molar ratio. D) Cleavage of mC5a by rScpA at 10:1 molar ratio. Duration of incubation in minutes is indicated above each lane. (TIF) [file ppat.1006493.s008.tif]

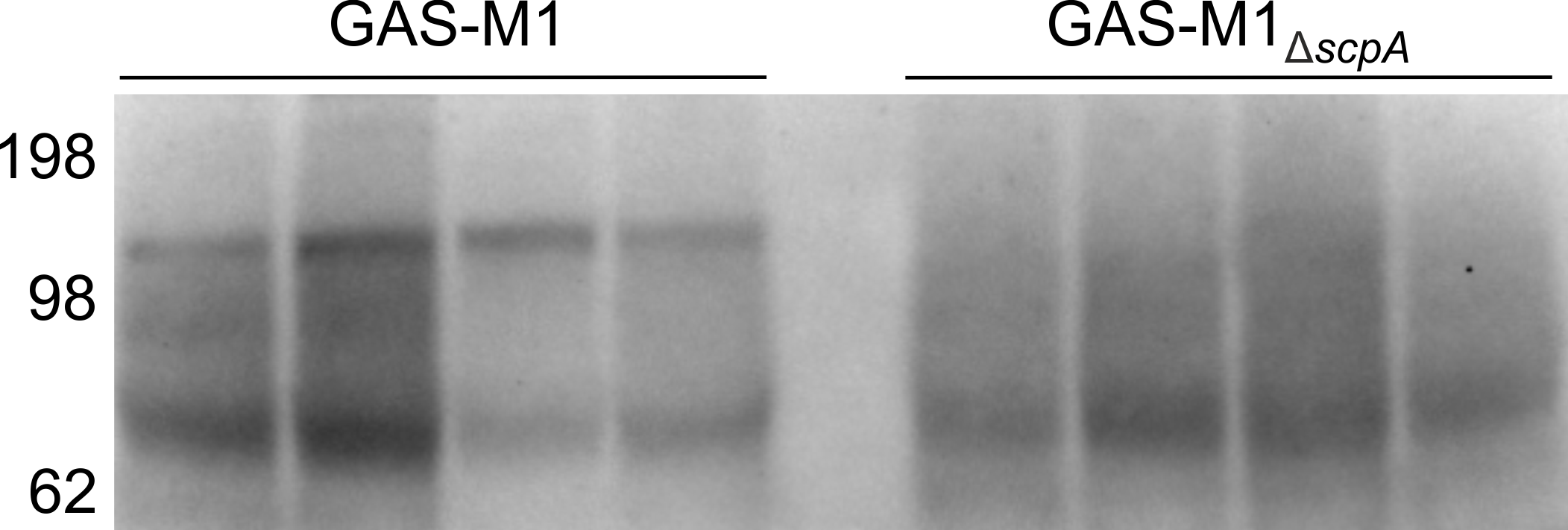

Supplement: S9 Fig — Expression analysis of ScpA in vivo. C57BL/6 mice were infected intra-muscularly with GAS-M1 or GAS-M1ΔscpA (n = 4/group). ScpA expression in 10 μg thigh tissue homogenate was compared between individual mice following immunoblot with anti-ScpA mouse serum. The band unique to GAS-M1 infected mice at 110 kDa is ScpA. (TIF) [file ppat.1006493.s009.tif]

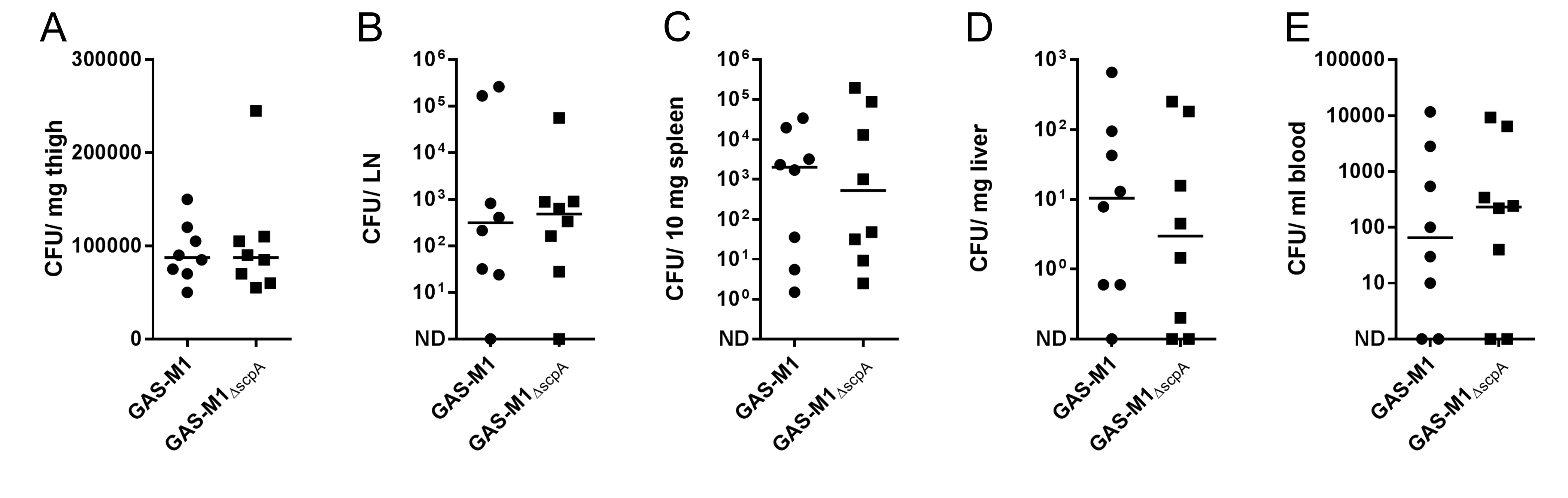

Supplement: S10 Fig — Characterization of GAS-M1 and GAS-M1ΔscpA dissemination from the site of infection in a murine model of soft tissue infection in wildtype C57BL/6 mice (n = 8/group) at 24 hour timepoint. Mice were infected intra-muscularly in the thigh. A) Bacteria at the site of infection, B) local spread to the draining inguinal lymph node, and subsequent systemic dissemination to C) spleen, D) liver and E) blood were assessed by quantitative culture. Each data point indicates one mouse and line depicts median value. (Mann Whitney U, * = p<0.05). (TIF) [file ppat.1006493.s010.tif]

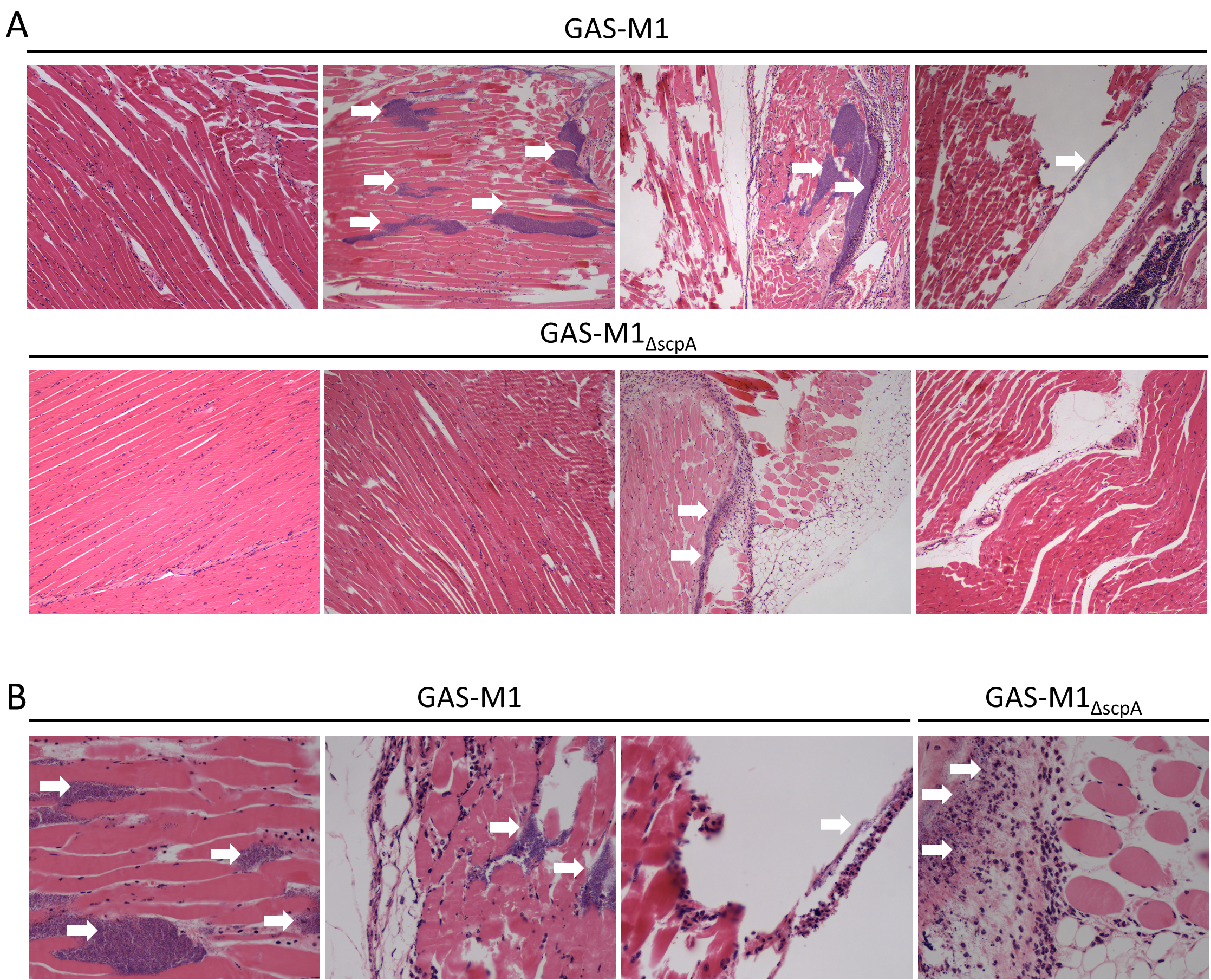

Supplement: S11 Fig — Haemotoxylin and eosin stained tissue sections of whole thigh obtained from C57BL/6 mice 3 hours after intra-muscular infection with GAS-M1 or GAS-M1ΔscpA (n = 4/group). A) Magnification x10. White arrows indicate regions with detectable GAS. Contiguous sections were Gram stained to aid detection of bacteria. B) Higher power (40x) images of sections with detectable bacteria from mice infected with GAS-M1 (n = 3/4) and GAS-M1ΔscpA (n = 1/4). White arrows indicate regions with detectable GAS. Note very limited but necrotic leukocyte infiltrate. (TIF) [file ppat.1006493.s011.tif]

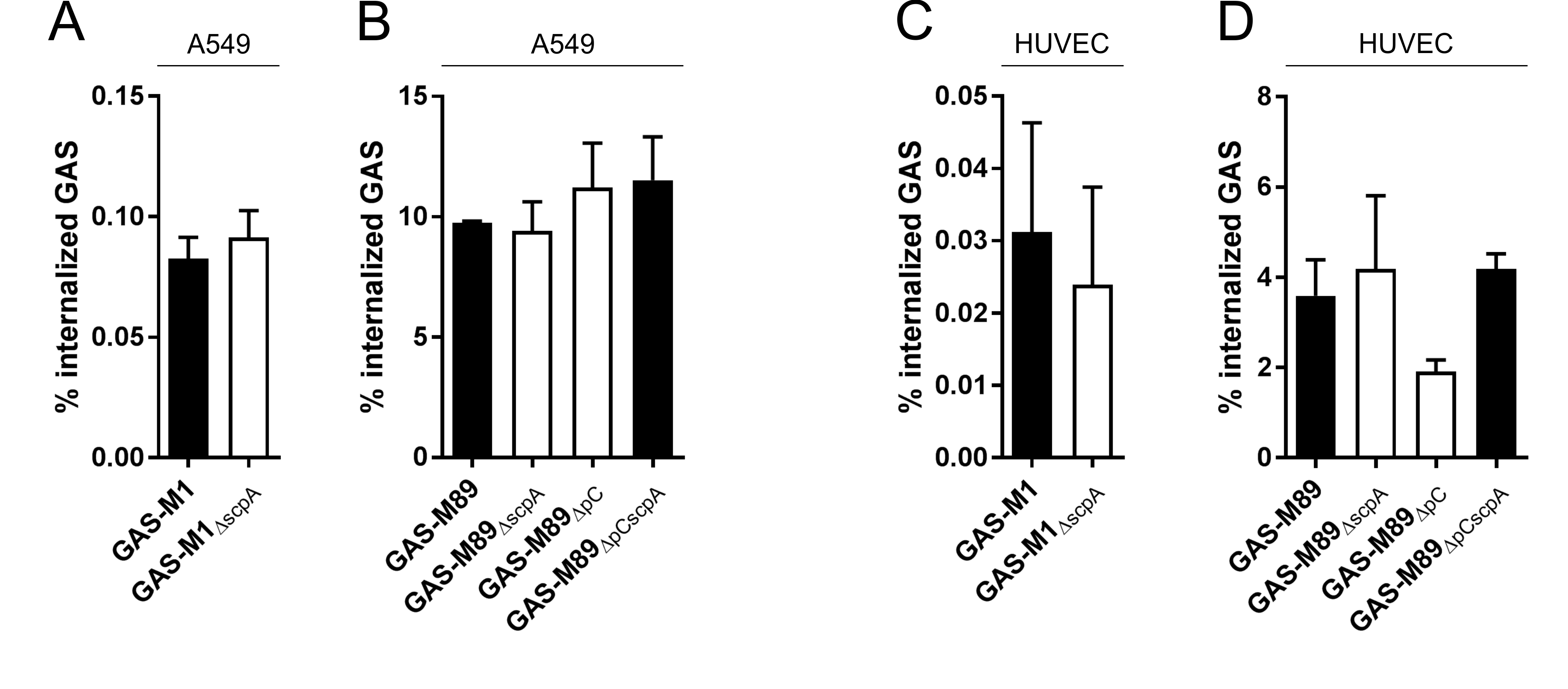

Supplement: S12 Fig — A+B) Internalization of A) GAS-M1 and B) GAS-M89 and isogenic ΔscpA strains by A549 epithelial cells was compared by quantitative culture (30 min incubation). Data represent mean+/-SD of 3 experimental replicates. C+D) Internalization of C) GAS-M1 and D) GAS-M89 and isogenic ΔscpA strains by primary human HUVEC was compared by quantitative culture (30 min incubation). Data represent mean+/-SD of 3 experimental replicates. (TIF) [file ppat.1006493.s012.tif]

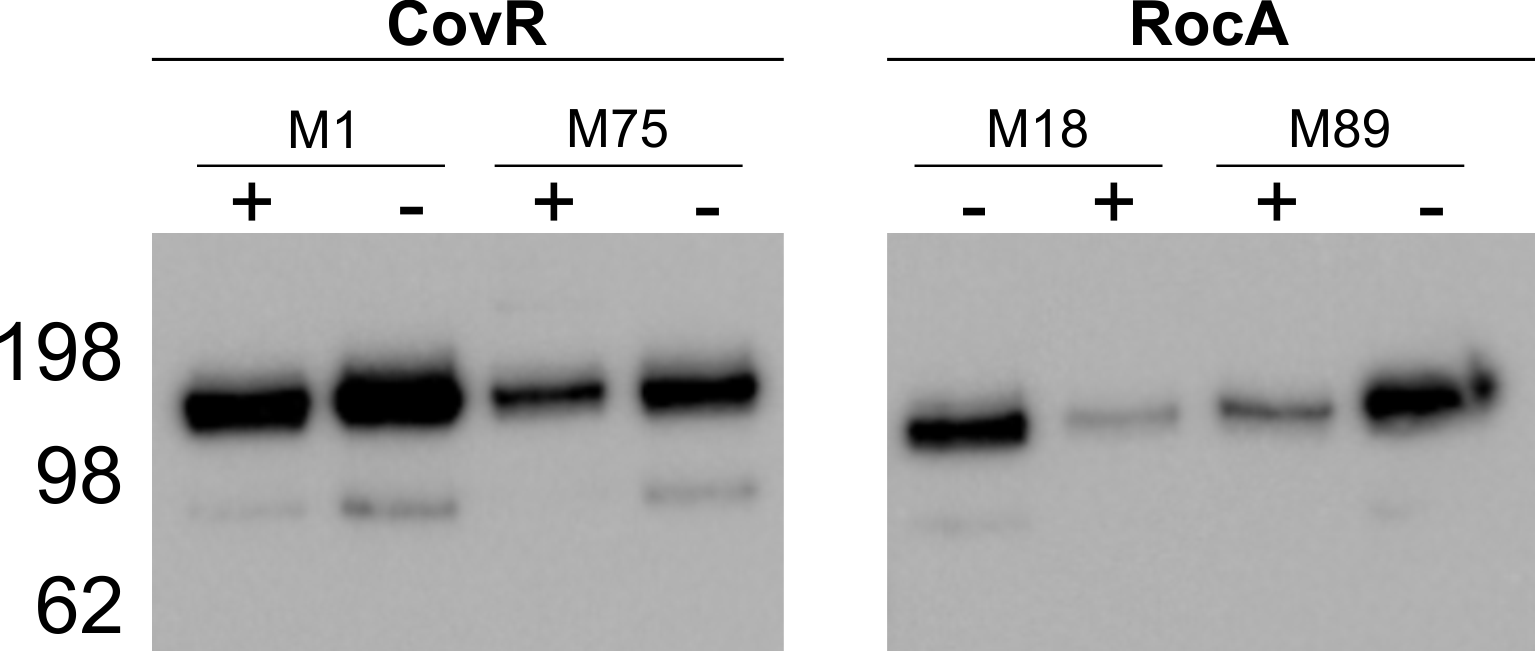

Supplement: S13 Fig — Western blot comparing expression of ScpA between pairs of isogenic strains expressing active or inactive CovR or RocA (strains listed in Table 1). ScpA expression in 1 μg bacterial cell wall extract was compared between isogenic strains following detection with anti-ScpA mouse serum. Functional CovR and RocA repressed expression of ScpA. (TIF) [file ppat.1006493.s013.tif]
