# Supplementary material for: Multi-functional mechanisms of immune evasion by the streptococcal complement inhibitor C5a peptidase
Source: PLoS Pathog. 2017 Aug 14;13(8):e1006493. doi: 10.1371/journal.ppat.1006493 (PMC5555575; doi:10.1371/journal.ppat.1006493)
Supplement: S1 Table — (PDF) [file ppat.1006493.s014.pdf]

	**Stimulant**	**% positive neutrophils (median,range)**	**gMFI (median, range)**
**Isotype control**	PBS	0.0335 (0.025)	8.115 (3.65)
	GAS-M1	0.165 (0.11)	22.5 (6.41)
	GAS-M1$_{\Delta scpA}$	0.071 (0.039)	26.6 (21)
**CD11b+**	PBS	98.75 (0.3)	79.9 (2)
	GAS-M1	99.2 (1.1)	121.5 (15)
	GAS-M1$_{\Delta scpA}$	98.8 (0.4)	204.5 (39)

S1 Table: Flow cytometry CD11b antibody specificity (4 technical replicates, representative of 3 donors)
